# Plasma proteins, cognitive decline, and 20‐year risk of dementia in the Whitehall II and Atherosclerosis Risk in Communities studies

**DOI:** 10.1002/alz.12419

**Published:** 2021-08-02

**Authors:** Joni V. Lindbohm, Nina Mars, Keenan A. Walker, Archana Singh‐Manoux, Gill Livingston, Eric J. Brunner, Pyry N. Sipilä, Kalle Saksela, Jane E. Ferrie, Ruth C. Lovering, Stephen A. Williams, Aroon D. Hingorani, Rebecca F. Gottesman, Henrik Zetterberg, Mika Kivimäki

**Affiliations:** ^1^ Department of Epidemiology and Public Health University College London London UK; ^2^ Department of Public Health Clinicum University of Helsinki Helsinki Finland; ^3^ Institute for Molecular Medicine Finland (FIMM) HiLIFE University of Helsinki Helsinki Finland; ^4^ Laboratory of Behavioral Neuroscience Intramural Research Program National Institute on Aging Baltimore Maryland USA; ^5^ Epidemiology of Ageing and Neurodegenerative diseases Université de Paris Paris France; ^6^ Division of Psychiatry University College London London UK; ^7^ Camden and Islington Foundation Trust London UK; ^8^ Department of Virology University of Helsinki and HUSLAB, Helsinki University Hospital Helsinki Finland; ^9^ Bristol Medical School (PHS) University of Bristol Bristol UK; ^10^ Functional Gene Annotation Institute of Cardiovascular Science University College London London UK; ^11^ SomaLogic, Inc. Boulder Colorado USA; ^12^ Institute of Cardiovascular Science University College London London UK; ^13^ British Heart Foundation Research Accelerator University College London London UK; ^14^ Health Data Research London UK; ^15^ Department of Neurology The Johns Hopkins University Baltimore Maryland USA; ^16^ Department of Neurodegenerative Disease and UK Dementia Research Institute University College London London UK; ^17^ Department of Psychiatry and Neurochemistry Institute of Neuroscience and Physiology The Sahlgrenska Academy University of Gothenburg Gothenburg Sweden; ^18^ Clinical Neurochemistry Laboratory Sahlgrenska University Hospital Mölndal Sweden

**Keywords:** cognitive decline, cohort study, dementia, longitudinal study, proteomics

## Abstract

**Introduction:**

Plasma proteins affect biological processes and are common drug targets but their role in the development of Alzheimer's disease and related dementias remains unclear. We examined associations between 4953 plasma proteins and cognitive decline and risk of dementia in two cohort studies with 20‐year follow‐ups.

**Methods:**

In the Whitehall II prospective cohort study proteins were measured using SOMAscan technology. Cognitive performance was tested five times over 20 years. Linkage to electronic health records identified incident dementia. The results were replicated in the Atherosclerosis Risk in Communities (ARIC) study.

**Results:**

Fifteen non‐amyloid/non‐tau–related proteins were associated with cognitive decline and dementia, were consistently identified in both cohorts, and were not explained by known dementia risk factors. Levels of six of the proteins are modifiable by currently approved medications for other conditions.

**Discussion:**

This study identified several plasma proteins in dementia‐free people that are associated with long‐term risk of cognitive decline and dementia.

## 1 BACKGROUND

Alzheimer's disease (AD) and related dementias pose an increasing challenge with considerable costs to the individual and to health and social care services.^1^ Amyloid beta (Aβ) and tau proteins have dominated pathophysiological research on dementia etiology,^2^ but to date prevention and treatment trials targeting these biomarkers have been unsuccessful.^3^, ^4^ In addition, longitudinal studies suggest that most cognitively normal amyloid‐positive people never develop clinical dementia.^5^ Given this and the high prevalence of mixed types of dementia in the general population, there is a need to expand research on early biomarkers for dementia beyond amyloid and tau.

Causes of dementia are increasingly thought to be systemic.^6^, ^7^ Recent development of scalable platforms allows simultaneous assessment of thousands of circulating proteins.^8^, ^9^ These may have the potential to identify novel drivers of dementia. For several reasons, circulating proteins are promising targets for biomarker and drug discovery. Proteins are regulators and effectors of biological processes and are also imprints of the effects of genes, the environment, age, current comorbidities, behaviors, and medications, all of which may affect dementia development.^10^ Proteins are also highly druggable as they can be targeted by monoclonal antibodies, small‐molecule drugs, or proteolysis‐targeting chimaeras.^11^, ^12^, ^13^, ^14^ Indeed, of all currently approved medications, ≈96% target proteins.^11^ Animal studies support a causal role of circulating plasma proteins in neurodegeneration. Plasma from young mice, via injection or through parabiosis (joining two animals so they share blood circulation), has been shown to restore memory and stimulate synaptic plasticity in the aged mouse hippocampus.^15^, ^16^, ^17^ In humans, multiple circulating proteins have been linked with dementia.^18^ However, human studies to date have been mainly cross‐sectional, based on small samples (N <1000), or lacked replication.

In this report from two large prospective cohort studies, the British Whitehall II and US Atherosclerosis Risk in Communities (ARIC) study, we used SOMAscan technology to examine 4953 plasma proteins as risk factors for cognitive decline and dementia. To determine the role of proteins in the early stages of neurodegeneration when dementia may still be preventable, we assessed plasma proteins in late middle age and followed subsequent cognitive decline and dementia over two decades. Our study design thus explicitly takes into consideration the long preclinical phase of dementia.

## 2 METHODS

### 2.1 Study design and participants

**FIGURE 1:**
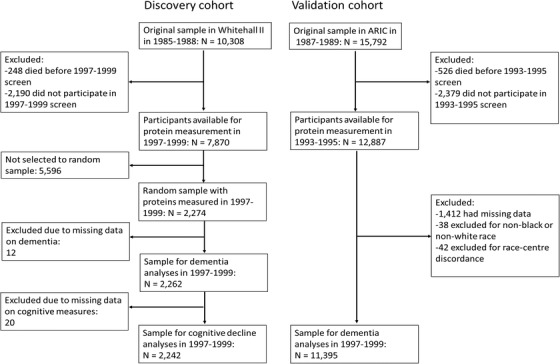
Flowchart of sample selection in discovery and validation cohorts. ARIC, the Atherosclerosis Risk in Communities study

We used the Whitehall II study as our discovery cohort and the ARIC study as the replication cohort (Figure 1). In 1985 to 1988, all civil servants aged 35 to 55 years based in 20 departments in London, UK, were invited to participate in the Whitehall II cohort study, and 73% (n = 10,308) agreed.^19^ Blood samples for proteomic analyses were collected from a random subsample of 2274 dementia‐free individuals in 1997 to 1999.^20^ Cognitive performance measurements were conducted at this and four subsequent clinical examinations in 2002 to 2004, 2007 to 2009, 2012 to 2013, and 2015 to 2016. Follow‐up started from 1997 to 1999 and ended at death, dementia, or in October 2019.

ARIC^21^ is a community‐based cohort study of 15,792 participants from four US communities: Washington County, Maryland; Forsyth County, North Carolina; northwestern suburbs of Minneapolis, Minnesota; and Jackson, Mississippi. Participants were aged 44 to 66 years at study entry in 1987 to 1989. Blood samples for protein analysis were drawn in 1993 to 1995 and analyzed for 11,395 individuals free of dementia. Prevalent dementia cases were identified from hospital discharge and by contacting participants annually via telephone or by administering a brief cognitive screener. Follow‐up for incident dementia ended at death, dementia, or in December 2017.

**RESEARCH IN CONTEXT**

1. **Systematic Review**: We searched PubMed for studies on plasma and serum proteome and dementia etiology, without language or date restrictions, up to March 11, 2021. Most of the identified studies had small (N <1000) sample size, lacked replication cohort, or were cross‐sectional and thus did not allow identification of proteins associated with early disease development when individuals are asymptomatic.
2. **Interpretation**: In this follow‐up of two large cohorts with a total of 13,657 participants, we identified and replicated 15 plasma protein biomarkers in asymptomatic individuals that were associated with cognitive decline and 20‐year risk of dementia. These proteins are involved in innate and adaptive immunity, blood‐brain barrier dysfunction, vascular pathology, and central insulin resistance providing evidence of the systemic pathogenesis of dementia.
3. **Future Directions**: Future research should examine whether the identified proteins are causal risk factors for dementia and whether they could provide clues for drug development or repurposing of existing medications.

### 2.2 Assessment of plasma proteins

In Whitehall II and ARIC, proteins were analyzed using the SOMAscan version 4 assay. The analyses used plasma ethylenediaminetetraacetic acid samples stored at –80°C. Earlier studies and Supplementary Methods in supporting information describe performance of the SOMAscan assay and the modified aptamer binding in detail.^22^, ^23^, ^24^ In brief, the assay uses a mix of thousands of slow off‐rate modified aptamers (SOMAmers). The aptamers bind to proteins in participants’ plasma samples and the specificity is ensured with a two‐step process analogous to a conventional immunoassay. Median intra‐ and inter‐assay coefficients of variation for SOMAscan version 4 are ≈5% and assay sensitivity is comparable to that of typical immunoassays, with a median lower limit of detection in the femtomolar range.^23^ The specificity of the aptamer reagents is good, has been tested in several ways,^10^, ^20^, ^24^, ^25^, ^26^, ^27^ and was also assessed in this study for protein hits using Olink Explore panel as a reference in a subset of 543 Whitehall II participants.

### 2.3 Cognitive testing

At least two complete measurements of the Whitehall II cognitive test battery were available for 2242 (99%) participants. The battery is sensitive to any decrease in cognitive performance and covers four domains: executive function, memory, and phonemic and semantic fluency (Supplementary Methods). Based on these measures, we constructed a global cognitive score by first standardizing the distribution of each test domain measured in follow‐up visits to the baseline score to create *z*‐scores with mean 0 and standard deviation (SD) 1. We then summed the domain specific scores at each phase and standardized the summary score to the baseline summary score; this approach minimizes measurement error inherent in individual tests.^28^

### 2.4 Dementia follow‐up

Whitehall II study participants were linked to the National Health Services (NHS) Hospital Episode Statistics (HES) database, and the British National mortality register using individual NHS identification numbers for linkage.^29^ The NHS provides nearly complete health‐care coverage for all individuals legally resident in the UK. Incident dementia was defined using the World Health Organization International Classification of Diseases, version 10 (ICD‐10) codes F00, F01, F03, G30, and G31 and ICD‐9 codes 290.0–290.4, 331.0–331.2, 331.82, and 331.9. Sensitivity and specificity of dementia assessment based on HES data are 0.78 and 0.92, respectively.^30^ We also conducted informant interviews and checked participants’ medications at each clinical examination for dementia‐related medication. In sensitivity analyses on dementia subtypes, those with prevalent atherosclerotic cardiovascular disease (coronary heart disease, heart failure, peripheral artery disease, or stroke) at the time of dementia diagnosis were classified as having vascular dementia and those without as having non‐vascular dementia.

In ARIC, incident dementia cases between 1993 and 2011 were identified by contacting participants annually via telephone or by administering a brief cognitive screener, and if applicable their caregivers completed a questionnaire. This information, supplemented by surveillance of dementia‐related hospital discharge and death certificate codes, was used to estimate the dementia onset date. Clinical and neuropsychological examinations to ascertain dementia cases were conducted in visits five (2011 to 2013) and six (2016 to 2017).^31^, ^32^ Using all available data, suspected cases were adjudicated by a committee of clinicians.

### 2.5 Measurement of baseline covariates

In the Whitehall II study, standard self‐administered questionnaires provided data on age, sex, ethnicity, socioeconomic status, education, medication, alcohol consumption, and smoking. Depressive symptoms were ascertained using the General Health Questionnaire (GHQ).^33^ ICD codes used to measure comorbidities (atrial fibrillation, coronary heart disease, depression, diabetes, heart failure, peripheral artery disease, and stroke) are provided in Supplementary Methods. Experienced clinical nurses measured body mass index (BMI) and systolic blood pressure and took blood samples for lipid and glucose measurements.^19^ Using DNA extracted from whole blood, a standard polymerase chain reaction (PCR) assay determined apolipoprotein E (*APOE*) genotype using the salting out method.^34^, ^35^ Two blinded independent observers read the genotype and any discrepancies were resolved by repeating the PCR analysis. In ARIC, baseline covariates included age, sex, and a combined race–study center variable, measured using standard operating protocols.

### 2.6 Statistical analysis

Proteins were transformed to a normal distribution by inverse rank‐based normal transformation. We used Spearman correlation to test protein‐protein correlations. To maximize power for selection of candidate protein predictors, we followed a two‐step protocol Figure S1a and S1b in supporting information and Supplementary Methods). The first step included a linear regression to examine associations between each protein and the cognitive decline slope derived from mixed‐effects linear regression of repeated cognitive assessments. The assumptions of linear regression were assessed by plotting the residuals in residuals versus fitted, normal Q‐Q, scale‐location, and residual versus leverage plots.^36^ We used false discovery rate (FDR) correction of 5% to select proteins from linear regression for the second step. This translated to a *P*‐value cut‐off of .002. In the second step, the proteins that survived FDR correction were analyzed in age‐, sex‐, and ethnicity‐adjusted Cox regression models^37^ with incident dementia as the outcome. *P*‐value = .05 was the cut‐off for statistical significance. The proportionality assumption in all Cox models was assessed with Schoenfeld residuals, log‐log plots, and time‐interaction coefficients^37^ and was not violated.

To examine reproducibility, proteins that were robustly associated with cognitive decline and dementia in the Whitehall II study were also analyzed in the ARIC study using dementia as an outcome. These Cox models were adjusted for age, sex and race‐study center. In sensitivity analyses conducted in the Whitehall II study, we ran Cox regression adjusted for age, sex, ethnicity, systolic blood pressure, total cholesterol, antihypertensive medication, smoking, diabetes, *APOE* genotype, BMI, alcohol consumption, education, and GHQ score to study the effect of confounders. No continuous covariate was categorized. We used this model to study whether the associations between proteins and dementia were attributable to reverse causation by excluding the first 10 years of follow‐up. To address potential survival bias, we conducted adjusted Fine and Gray competing risk analysis^38^ with dementia and death as outcomes. The effect of comorbidities on the protein–dementia associations was studied with time‐varying covariates and the effect of missing data was examined with multiple imputation (Supplementary Methods). To minimize bias due to ethnic admixture in ARIC, we performed a sensitivity analyses after excluding non‐White participants from the analyses.

For identified and replicated proteins, we searched the Genotype‐Tissue Expression (GTEx);^39^ Human Protein Atlas;^40^, ^41^ Database for Annotation, Visualization and Integrated Discovery (DAVID);^42^, ^43^ UniProt;^44^ and ChEMBL^45^ databases to characterize protein expression profiles, their cellular localization, and drugs that can target them. We used statistical software R (3.6.0) and Stata (version 16.1 MP; Stata Corp) for all analyses.

## 3 RESULTS

Mean age of the 2274 Whitehall II participants was 56.1 (SD 5.9), and 1653 (73.0%) were men (Table 1). During a mean follow‐up of 20.4 years, 106 individuals developed dementia. After FDR correction of 5%, 246 of the 4953 proteins were associated with an increased rate of cognitive decline. None of the amyloid‐, tau‐, or neurofilament‐related proteins were associated with accelerated cognitive decline after FDR correction (Table S1 in supporting information).

**TABLE 1:** Participant characteristics at the time of blood collection for protein measurement in the Whitehall II study and the Atherosclerosis Risk in Communities study

Characteristic, n (%) or mean (SD)	Whitehall II N = 2274	ARIC N = 11,395
Demographic variables		
Age, mean (SD)	56.1 (5.9)	60.2 (5.7)
Men, No. (%)	1653 (73.0)	5190 (45.6)
White, No. (%)	2089 (92.3)	8991 (78.9)
Education, No. (%)		
Less than high school	522 (31.1)	2311 (20.3)
High school/vocational	427 (25.4)	4813 (42.3)
College/graduate/ professional	731 (43.5)	4255 (37.4)
Apolipoprotein E ε4 alleles, No. (%)		
0	1432 (73.4)	7678 (67.4)
1	469 (24.1)	3358 (29.5)
2	49 (2.5)	359 (3.2)
Physiological and lab variables, mean (SD)		
Body mass index, kg/m^2^	26,6 (4,1)	28.5 (5.6)
Total cholesterol, mg/dL	232.1 (40.4)	208.8 (38.7)
Cardiovascular risk factors, No. (%)		
Hypertension	286 (12.6)	4686 (41.3)
Diabetes mellitus	13 (0.6)	1806 (15.9)
Cigarette smoking, current	204 (9.1)	2042 (18.0)
Mean follow‐up time (SD)	20.4 (3.2)	17.7 (6.1)
Median follow‐up time (IQR)	21.5 (21.0, 22.1)	20.0 (14.1, 22.4)

Abbreviations: ARIC, Atherosclerosis Risk in Communities study; IQR, interquartile range; SD, standard deviation.

Notes: Values are displayed as means (SD) for continuous variables, frequencies (column percentages) for categorical variables, and median (IQR) for follow‐up time.

**FIGURE 2:**
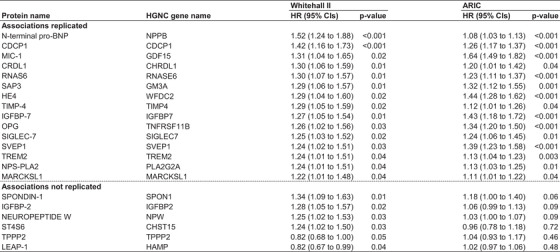
Proteins associated with dementia in Whitehall II and ARIC cohorts. HR, hazard ratio; 95% CI, 95% confidence interval; ARIC, the Atherosclerosis Risk in Communities study; SD, standard deviation; FDR, false discovery rate of 5%; Protein names: N‐terminal pro‐BNP, N‐terminal pro‐BNP; CDCP1, CUB domain‐containing protein 1. MIC‐1, growth/differentiation factor 15. CRDL1, Chordin‐like protein 1; RNAS6, ribonuclease K6; SAP3, ganglioside GM2 activator; HE4, WAP four‐disulfide core domain protein 2; TIMP‐4, metalloproteinase inhibitor 4; IGFBP‐7, insulin‐like growth factor‐binding protein 7; OPG, tumor necrosis factor receptor superfamily member 11B; Siglec‐7, sialic acid‐binding Ig‐like lectin 7; SVEP1, Sushi, von Willebrand factor type A, EGF and pentraxin domain‐containing protein 1; TREM2, triggering receptor expressed on myeloid cells 2; NPS‐PLA2, phospholipase A2, membrane associated; MARCKSL1, MARCKS‐related protein; Spondin‐1, spondin‐1; IGFBP‐2, insulin‐like growth factor‐binding protein 2; Neuropeptide W, neuropeptide W; ST4S6, Carbohydrate sulfotransferase 15; TPPP2, tubulin polymerization‐promoting protein family member 2; LEAP‐1, hepcidin

Of the 246 proteins, 21 were also associated with incident dementia in the Whitehall II study (Figure 2). Thus, replication analyses in ARIC were based on these 21 proteins. In ARIC, 1942 of the 11,395 participants developed dementia during a mean follow‐up of 17.4 years. Of the 21 proteins associated with cognitive decline and dementia in the Whitehall II study, 15 (71%) were also associated with dementia in the ARIC cohort. Levels of all 15 proteins were higher in individuals who developed dementia (Figures S2‐S4 in supporting information). Hazard ratios for a 1 SD higher level in each of the 15 proteins ranged from 1.08 to 1.64 in age‐, sex‐, and ethnicity/race‐adjusted analyses. A cognitive domain–specific analysis showed that phonemic fluency and executive functioning were the main drivers of the associations between the 15 proteins and a higher rate of cognitive decline (Table S2‐S3 in supporting information).

**FIGURE 3:**
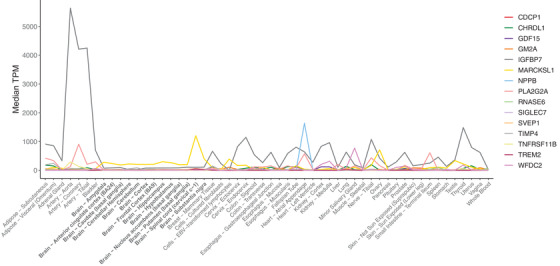
Expression profile of the genes coding the 15 proteins associated with rate of cognitive decline and dementia derived from Genotype‐Tissue Expression (GTEx) database. Y‐axis shows the extent to which these genes are expressed by the organs and tissues listed in X‐axis. For clarity, tissues in the brain are marked in bold. EBV, Ebstein‐Barr virus; TPM, transcripts per million

Of the 15 identified and replicated proteins, 14 (N‐terminal pro‐BNP, CDCP1, MIC‐1, CRDL1, RNAS6, SAP3, HE4, TIMP‐4, IGFBP‐7, OPG, SVEP1, TREM2, NPS‐PLA2, MARCKSL1) were secreted and one (SIGLEC‐7) was a cell membrane protein. They were mostly expressed in tissues other than the brain (Figure 3 and Table S4 in supporting information). The proteins were related to one or more biological systems that are relevant to dementia etiology: activation of the immune system, blood‐brain barrier (BBB) breakdown, vascular pathology, and central insulin resistance (Table 2). For six proteins, a medication that can influence the protein's function (NPS‐PLA2, CDCP1, MIC‐1, and IGFBP‐7) or reduce its levels (N‐terminal pro‐BNP, and OPG) is available. The medications are used to treat diabetes, cancer, and inflammatory or cardiovascular diseases and all except the one on MIC‐1 have passed phase III trials (Table 3).

**TABLE 2:** Association between 15 proteins and dementia‐related pathologies

	Association with				
Protein	Immune system	BBB dysfunction	Vascular pathology	Central insulin resistance	Previous studies with dementia outcome
N‐terminal pro‐BNP	No	No	Yes	No	Prospective cohort^56^, ^57^, ^103^, ^104^
CDCP1	Yes	Yes	No	No	None
MIC‐1	Yes	Yes	Yes	No	Case control^49^, ^50^
CRDL1	No	No	Yes	No	None
RNAS6	Yes	No	No	No	None
SAP3	No	Yes	Yes	No	Case‐control^27^
HE4	Yes	Yes	Yes	No	None
TIMP‐4	Yes	Yes	Yes	No	Case‐control^51^
IGFBP‐7	No	No	Yes	Yes	Case‐control^48^
OPG	Yes	Yes	Yes	No	Case‐control^52^
SIGLEC‐7	Yes	No	No	No	None
SVEP1	Yes	Yes	Yes	No	None
TREM2	Yes	No	No	No	Prospective cohort^55^
NPS‐PLA2	Yes	No	Yes	No	Case‐control^54^
MARCKSL1	Yes	Yes	No	No	None
Total N of proteins	11	8	10	1	8

Notes: In the first four columns, "Yes" indicates that the protein has been linked to the pathology and "No" indicates no evidence of such a link is available. Row "Total" provides the total number of protein‐pathology associations for each dementia‐related pathology. The fifth column provides reference and type of evidence linking these proteins to dementia risk.

Abbreviation: BBB, blood‐brain barrier.

**TABLE 3:** Drugs that can influence currently identified proteins associated with dementia, their potential mechanisms of action, and current indications

Protein	Medication	Action	Phase passed	Indications
CDCP1	Itolizumab^118^	Prevents CDCP1 binding to CD6 and down regulates T cell activation and infiltration. It also reduces synthesis of pro‐inflammatory cytokines reducing T cell infiltration at sites of inflammation.	III	Psoriasis
NPS‐PLA2	Varespladib and Varespladib Methyl^117^	Inhibits arachidonic acid pathway in inflammation by inhibiting NPS‐PLA2 activity and subsequent leukocyte activation.	III	Atherosclerotic cardiovascular diseases, inflammatory diseases, snake venom antidote
IGFBP‐7	Intranasal insulin, metformin, and GLP‐1 receptor agonists^113^	Insulin nasal spray restores central insulin levels that may be downregulated by elevated IGFBP‐7 levels. Metformin acts as insulin sensitizer and GLP‐1 agonists stimulate insulin secretion.	III	Cognitive decline and Alzheimer´s disease
N‐terminal pro‐BNP	Antihypertensive medications^103^	Reduce N‐terminal pro‐BNP levels in circulation by reducing atrial and ventricular overload.	III	Hypertension
OPG	Atorvastatin, metformin, pioglitazone, rosiglitazone^88^, ^119^, ^120^	Reduce OPG levels possibly by reducing inflammation and by stabilizing atherosclerotic plaques.	III	Atherosclerotic cardiovascular diseases, diabetes
MIC‐1	Monoclonal antibody CTL‐002^121^	Neutralizes MIC‐1	‐	Advanced cancer

A series of sensitivity analyses based on the Whitehall data suggest that our findings are robust. The decrease in hazard ratios was between 0.00 and 0.07 for the 15 protein–dementia associations after multivariable adjustment for age, sex, ethnicity, education, systolic blood pressure, antihypertensive medication, total cholesterol, BMI, alcohol consumption, smoking, diabetes, GHQ score, and *APOE* status; after adjusting comorbidities (atrial fibrillation, coronary heart disease, depression, diabetes, heart failure, peripheral artery disease, and stroke, all treated as time‐varying covariates); after reducing the possibility of reverse causation bias by excluding dementia cases that occurred during the first 10 years of follow‐up; after taking into account the competing risk of death; and after imputation of missing covariates (Table S5 in supporting information). The results did not change markedly after excluding non‐White participants in the ARIC study (Table S6 in supporting information). There were no sex differences in observed protein–dementia associations (range of *P*‐values for interaction from 0.11 to 0.99). Associations between standard risk factors and dementia were similar to those reported in recent meta‐analyses,^46^, ^47^ suggesting that Whitehall is not an exceptional cohort study (Table S7 in supporting information).

There were no strong correlations between the 15 proteins (all correlations ≤|0.53|, Figure S5 in supporting information). Nine proteins had higher HR for non‐vascular and six for vascular dementia but the differences were not statistically significant and could not be studied reliably due to lack of power (Table S8 in supporting information).

Correlations between the 11 proteins measured with SOMAscan version 4 and Olink Explore platform ranged between 0.46 and 0.87 (Table S9 in supporting information). Previous studies also suggest the measurement of proteins based on SOMAScan aptamers is reliable and specific (Table S10 in supporting information). Of the 15 proteins identified by aptamers, eight (N‐terminal pro‐BNP, MIC‐1, HE4, TIMP‐4, IGFBP‐7, SVEP1, TREM2, and NPS‐PLA2) have been previously cross‐validated with more than one method including mass spectrometry, immunoassay, Olink protein panel, and by identification of genetic variants influencing the concentration of the protein measured using the Somalogic platform in the vicinity of the encoding gene (i.e., *cis* protein quantitative trait loci data). Six other proteins have been cross‐validated using one method, four (CDCP1, RNAS6, OPG, and Siglec‐7) using *cis* protein quantitative trait loci data, one (CRDL1) using mass spectrometry, one (SAP3) indirectly from an association between SAP3 and AD observed using mass spectrometry, while one of the 15 proteins (MARCKSL1) remains unvalidated.

## 4 DISCUSSION

To our knowledge, this is the largest proteome‐wide study with replicated results on long‐term associations between plasma proteins, cognitive decline, and risk of dementia to date. We identified 15 plasma proteins that were associated with increased rate of cognitive decline and an increased 20‐year risk of dementia in the British Whitehall II study. Associations with dementia were replicated in an independent US cohort study (ARIC); were robust to adjustments for known dementia risk factors; and were not explained by competing risk of death, cardiometabolic comorbidities, or reverse causation bias. The 15 proteins, of which 14 were secreted and 1 was a cell membrane protein, were mostly expressed outside the central nervous system suggesting that they relate to systemic processes that increase dementia risk.

Our findings are consistent with previous research. Proteins SAP3, NPS‐PLA2, IGFBP‐7, MIC‐1, TIMP‐4, and OPG have been reported to be associated with dementia in case‐control studies,^27^, ^48^, ^49^, ^50^, ^51^, ^52^, ^53^, ^54^ and TREM2 and N‐terminal pro‐BNP in prospective cohort studies,^55^, ^56^, ^57^ with all associations in the same direction as in this study. The increased dementia risk in individuals with high levels of plasma SVEP1, HE4, CDCP1, SIGLEC‐7, MARCKSL1, CRDL1, or RNAS6 is a novel finding. Our results also provide new evidence that midlife circulating TREM2 predicts dementia at older ages.

This study did not sytematically examine processes underlying the associations between proteins and dementia. However, the observed associations are biologically plausible as there are several mechanisms that could link the 15 proteins to dementia pathologies, including immune dysfunction, BBB dysfunction, vascular damage, and central insulin resistance. More specifically, hyper‐activation of the innate and adaptive immune system can cause endothelium damage, neuroinflammation, and amyloid and tau accumulation in the brain (Figure S6a and Sb in supporting information).^58^, ^59^, ^60^, ^61^ SVEP1 contributes to this process by activating the complement system, which, if prolonged, is detrimental to endothelial cells^62^ and may contribute to hippocampal synapse loss observed in dementia.^63^, ^64^ SIGLEC‐7 is an inhibitory receptor in natural killer cells that recognizes “self” antigens;^65^ its cleavage to plasma is thought to mark uncontrolled inflammation.^66^ Increased plasma concentrations of other proteins could represent responses to pathogens (e.g., RNAS6^67^, ^68^ and HE4),^69^ or be part of the increased immune response (e.g., NPS‐PLA2, TREM2, MIC‐1, OPG, TIMP‐4, SVEP1, MARCKSL1, and CDCP1).^49^, ^52^, ^53^, ^55^, ^66^, ^70^, ^71^, ^72^, ^73^, ^74^, ^75^, ^76^, ^77^, ^78^, ^79^

Degeneration of the BBB provides a further potential mechanism underlying the observed protein–dementia associations. The subsequent passage of toxins and proteins into the central nervous system contributes to reduced neuronal plasticity, activation of microglia, disruptions in lipid metabolism, increased neuroinflammation, amyloid and tau accumulation, and neurodegeneration (Figure S6c).^6^, ^7^, ^71^, ^72^, ^80^, ^81^, ^82^, ^83^ Elevated SAP3 levels, also found in the cerebrospinal fluid of AD and Parkinson's disease patients,^27^, ^84^ have been linked to the degeneration of pericytes that are crucial for the function of BBB;^85^, ^86^ increased MIC‐1 and OPG are associated with endothelial dysfunction;^87^, ^88^, ^89^ increased CDCP1 with tight junction degeneration;^90^, ^91^, ^92^ and MARCKSL1 and SVEP1 with endothelia integrity.^93^, ^94^, ^95^

Proteins TIMP‐4, SAP3, NPS‐PLA2, OPG, and MIC‐1 have been linked to vascular damage caused by atherosclerosis and thrombosis (Figure S6d).^87^, ^89^, ^96^, ^97^, ^98^, ^99^, ^100^, ^101^, ^102^ N‐terminal pro‐BNP is elevated in response to atrial and ventricular overload,^103^ and in plasma and the central nervous system, it can inhibit the activity of neprilysin, an enzyme that reduces Aβ levels.^104^ Elevated N‐terminal pro‐BNP levels are related to increased risk of vascular and AD dementia,^56^, ^57^, ^105^, ^106^ SVEP1 promotes thrombosis,^107^, ^108^ and HE4 and CRDL1 contribute to ischemia‐related abnormal angiogenesis^109^, ^110^, ^111^ observed in vascular and AD dementia.^6^, ^112^ At least 1 of the 15 proteins may contribute to central insulin resistance (Figure S6e)^113^ as IGFBP‐7 reduces free plasma insulin levels and also prevents free insulin from binding to its receptor,^114^, ^115^ both linked to reduced insulin responsiveness.^116^

For some of the identified proteins, approved protein‐modifying medication already exists, but they are for the treatment of conditions other than dementia. Varespladib, currently used to treat cardiovascular and inflammatory diseases, inhibits the arachidonic acid inflammatory pathway by inhibiting NPS‐PLA2 activity.^117^ Itolizumab, used for psoriasis, prevents CDCP1 from binding to CD6, leading to downregulation in T‐cell–mediated inflammation.^118^ Glucose‐lowering and statin medications reduce OPG levels.^88^, ^119^, ^120^ Intranasal insulins^116^ could counteract high levels of insulin‐binding IGFBP‐7. Antihypertensive medications reduce plasma N‐terminal pro‐BNP levels.^103^ Furthermore, a monoclonal antibody that neutralizes MIC‐1 is currently being studied in a phase‐1 cancer trial.^121^ Whether these drugs could play a role in future prevention or treatment of dementias (i.e., drug repurposing) needs to be investigated in genetic, systems biology, and intervention studies.

Our study has some important strengths. SOMAscan is the largest protein panel available for large‐scale studies covering proteins that enter the bloodstream by purposeful secretion to orchestrate biological processes, by cleavage from cell membranes, or by leakage from intracellular space that inform cell injury rather than biological causality.^122^ In a subsample of Whitehall participants, the specificity of aptamers measuring 11 of the 15 dementia‐related proteins was supported by moderate to high inter‐assessment correlations between SOMAscan and Olink protein panels. In addition, the specificity of aptamers measuring 14 of the 15 proteins has been confirmed using mass spectrometry or orthogonal strategies.^10^, ^24^, ^25^, ^26^, ^27^ This suggests our results are unlikely to be biased by measurement error due to aptamer binding to unintended target proteins. Replication of the results in an independent study with different dementia ascertainment methods, dementia conversion rate, settings, and participant characteristics supports the validity and generalizability of the findings. We were able to confirm the associations in analyses excluding dementia cases diagnosed during the first 10 years of follow‐up. This finding is consistent with the possibility that we identified proteins that play a role in the early stages of neurodegeneration when dementia may still be preventable rather than those which only mark plasma changes caused by advanced preclinical disease.

The study has several limitations. First, SOMAscan covers only ≈5000 proteins of all the nearly 20,000 proteins identified by the Human Proteome Project from genomic open reading frames.^122^ In addition, our assessment was not optimized for the measurement of amyloid‐, tau‐, and neurofilament‐related proteins in plasma, which may explain that none of these proteins were significantly associated with cognitive decline and dementia. As the sample size was limited in our discovery cohort, we may have missed other important associations, such as the one between plasma apoE and AD.^123^, ^124^, ^125^ Lack of repeat data on proteins inhibited us to assess stability of proteins over time. However, the absence of effect attenuation in protein–dementia associations during the 20‐year follow‐up suggests that SOMAscan protein measurements were stable.

Second, our discovery study, based on electronic health records, missed cases of dementia not diagnosed or treated in UK hospitals. However, previous studies on dementia ascertainment based on electronic health records in the UK suggest that our ascertainment method is valid for a study of risk factor–dementia associations.^30^ Further limitations include the use of semi‐quantitative protein data that cannot determine clinically useful concentrations, limited numbers of vascular and non‐vascular dementia to study these subtypes more extensively, and the lack of information on detailed dementia subtypes based on brain imaging or cerebrospinal fluid biomarkers.

In conclusion, using large‐scale testing of plasma proteins as long‐term risk factors for cognitive decline and dementia, our results support the hypothesis that early systemic processes may drive dementia development. The protein–dementia associations identified and replicated were robust and plausible, potentially involving innate and adaptive immunity, BBB dysfunction, vascular pathology, and central insulin resistance, all of which can contribute to amyloid and tau deposition, or pathologies that characterize vascular dementia. As observational studies cannot determine causal associations, the present findings should be considered hypothesis generating. Further research is needed to determine whether the observed protein‐dementia associations are causal rather than driven by other biomarkers that have shared downstream effects with the proteins or attributable to compensatory mechanisms that modify protein levels in those who will later develop dementia.

## CONFLICTS OF INTEREST

In this academic–industry partnership project, academic collaborators generated the hypothesis and study design and SomaLogic, Inc. provided expertise in plasma proteins and funded the SOMAscan assays. Joni V. Lindbohm and Pyry N. Sipila have received personal lecture fees from the University of Helsinki. Nina Mars reports no conflicts of interest. Keenan A. Walker reports personal lecture fee from the Boston University Medical Center and holds the Programming Chair at the National Academy of Neuropsychology. Eric J. Brunner reports Osaka University research capacity building grant paid to employer. Archana Singh‐Manoux reports no conflicts of interest. Gill Livingston reports no conflicts of interest. Kalle Saksela reports no conflicts of interest. Jane E. Ferrie reports no conflicts of interest. Ruth C. Lovering reports personal lecture fees from the University College London, funding from a COST action grant, and is a member of the executive committee for the International Society of Biocuration (a voluntary role and no payment has been or will be made). Stephen A. Williams is employee of SomaLogic Inc., which has a commercial interest in the results and co‐inventor on multiple patents for specific proteomic models of disease. None of these patents relate to dementia (the topic of the manuscript). Aroon D. Hingorani reports no conflicts of interest. Rebecca F. Gottesman reports personal lecture fees for speaking at University of Michigan grand rounds, University of Alabama McKnight lecture, and the American College of Cardiology conference. Rebecca F. Gottesman is the secretary for the American Neurological Association. Henrik Zetterberg reports that he has served on the scientific advisory boards for Denali, Roche Diagnostics, Wave, Samumed, Siemens Healthineers, Pinteon Therapeutics, and CogRx; has given lectures in symposia sponsored by Fujirebio, Alzecure, and Biogen; and is a co‐founder of Brain Biomarker Solutions in Gothenburg AB (BBS), which is a part of the GU Ventures Incubator Program. Henrik Zetterberg is also the chair of the Alzheimer's Association Global Biomarker Standardization Consortium and the Alzheimer's Association Biolfluid‐Based Biomarker Professional Interest Area. Mika Kivimaki reports no conflicts of interest.

## DATA SHARING STATEMENT

Data, protocols, and other metadata of the Whitehall II and ARIC studies are available to the scientific community. Please refer to the data sharing policies of these studies. Pre‐existing data access policies for Whitehall II and ARIC studies specify that research data requests can be submitted to each steering committee; these will be promptly reviewed for confidentiality or intellectual property restrictions and will not unreasonably be refused. Individual‐level patient or protein data may further be restricted by consent, confidentiality, or privacy laws/considerations. These policies apply to both clinical and proteomic data. Detailed information on data sharing can be found here: https://www.ucl.ac.uk/epidemiology‐health‐care/research/epidemiology‐and‐public‐health/research/whitehall‐ii/data‐sharing

## Supporting information

Supporting Information
