# Supplementary material for: Plasma proteins, cognitive decline, and 20‐year risk of dementia in the Whitehall II and Atherosclerosis Risk in Communities studies
Source: Alzheimers Dement. 2021 Aug 2;18(4):612–24. doi: 10.1002/alz.12419 (PMC9292245; doi:10.1002/alz.12419)

Supplement

Contents

Supplementary Methods

Supplementary Figures 1a and b. Sample size needed to detect a range of beta coefficients using linear regression or Cox proportional hazards model.

Supplementary Table 1. Beta for 1 standard deviation increase in amyloid, tau, and neurofilament related protein levels and cognitive decline.

Supplementary Figure 2. Associations between each of the 4953 proteins and rate of cognitive decline by –log10 p-values and beta coefficient.

Supplementary Figure 3. Associations between each of the 4953 proteins and dementia by –log10 p-values and beta coefficient.

Supplementary Figure 4. Violin plots describing differences in standardized protein levels by dementia status.

Supplementary Table 2. Associations between the cognitive decline slope and a SD increase in the 15 proteins that survived replication.

Supplementary Table 3. Associations between the decline slopes in all cognitive domains measured and a SD increase in the 15 proteins that survived replication.

Supplementary Table 4. Protein expression levels in the brain tissue for 15 proteins that survived replication.

Supplementary Table 5. Sensitivity analyses for the 15 proteins associated with both cognitive decline and dementia in the Whitehall II study.

Supplementary Table 6. Sensitivity analyses including all or only white participants of the ARIC study and the 15 proteins associated with both cognitive decline and dementia in the Whitehall II study.

Supplementary Table 7. Associations between common dementia risk factors and dementia in Whitehall II, and estimates from most recent meta-analyses.

Supplementary Figure 5. Between protein correlations.

Supplementary Table 8. Sensitivity analyses of dementia subtypes for the 15 proteins associated with both cognitive decline and dementia in the Whitehall II study.

Supplementary Table 9. Spearman correlations between plasma proteins measured with SOMAscan version 4 and Olink Explore platform.

Supplementary Table 10. Validation of Somalogic aptamer-based protein measurements.

Supplementary Figure 6. Hypothesize dementia-related pathologies for 15 proteins associated with cognitive decline rate and dementia risk.

Supplementary Methods

Assessment of plasma proteins

In Whitehall II and ARIC, proteins were analyzed using the SOMAscan version 4 assay. The analyses used plasma samples measured in 1995/1997 and stored in 0.25 mL aliquots at −80◦C. Earlier studies describe performance of the SOMAscan assay and the modified aptamer binding in detail.[22-24] In brief, the assay uses a 96-well plate including a mix of thousands of slow off-rate modified aptamers (SOMAmers). The SOMAmers are labeled with biotin and a 5′ fluorophore, photocleavable linker and immobilized on streptavidin-coated beads through biotin–streptavidin interaction. Participants´ plasma samples were diluted and added to each well, which resulted in formation of cognate and non-specific SOMAmer-protein complexes. After the unbound proteins were washed away, captured proteins were labelled with biotin. By using ultraviolet light photocleavage, the SOMAmer-protein complexes were released from the beads and incubated in a well containing dextran sulfate and unlabeled polyanionic competitor. This step separates the non-specific, fast off-rate SOMAmer-protein complexes from slow off-rate cognate and thus intended complexes and can be viewed as a second element of specificity analogous to the effect of adding a second antibody in a conventional immunoassay.

Cognate SOMAmer-protein complexes were then recaptured on a second set of streptavidin coated beads using biotin labelled proteins and additionally washed to further remove non-specific SOMAmer-protein interactions. In the next step, denaturing buffer released the SOMAmers from the proteins. The SOMAmers were then hybridized to complementary sequences on a DNA microarray chip and quantified by fluorescence for readout. In the SOMAscan assay, fluorescence intensity for each SOMAmer is related to the relative availability of the three-dimensional shape-charge epitope on each protein (the binding site of the SOMAmer reagent) in the original sample. This describes the concentration of each protein but can also reflect the shape of the protein (that may itself reflect a genetic variant or modification) and any potential circulating competitor (physiologic or a therapeutic antibody) to some extent. However, the specificity of the modified aptamer reagents is good and has been tested in several ways.[10,20,25-28] Median intra- and inter-assay coefficients of variation for SomaScan version 4 are ~5% and assay sensitivity is comparable to that of typical immunoassays, with a median lower limit of detection in the femtomolar range.[23]

Cognitive testing

The Whitehall II cognitive test battery covered four domains: executive function, memory, phonemic-, and semantic fluency. Executive function was assessed with the Alice Heim 4-I test that comprises a series of 65 verbal and mathematical reasoning items of increasing difficulty.[128] It tests inductive reasoning, measuring the ability to identify patterns and infer principles and rules, and has time limit of 10 minutes. A 20-word free recall test assessed memory. Participants were presented a list of one or two syllable words at two-second intervals and were then asked to recall in writing as many of the words as possible in any order within two minutes. In assessment of phonemic and semantic fluency[129] participants were asked to recall in writing as many words beginning with “s” (phonemic fluency) and as many animal names (semantic fluency) as they could. Time limit for this section was one minute per fluency area.

Based on these measures, we created a global cognitive score by first standardizing the distribution of each test domain measured in follow-up visits to the baseline score to create z-scores with mean 0 and SD 1. We then summed the domain specific scores at each phase and standardized the summary score to the baseline summary score; this approach minimizes measurement error inherent in individual tests.[29] After dementia diagnosis, participants were rarely able to complete the cognitive tests and for this reason, their global test score was set to -3SD at the phase following the dementia diagnosis. Based on the global scores we derived a cognitive decline slope for each participant and used this as the outcome.

Comorbidities

The World Health Organization International Classification of Diseases (ICD) codes were retrieved from the National Health Services (NHS) Hospital Episode Statistics (HES) database records and from mortality registers using individual NHS identification numbers for linkage.[30] The NHS provides nearly complete comprehensive health care coverage for all individuals legally resident in the UK. Cardiovascular disease diagnoses as ascertained via HES has shown 70% sensitivity and 96% specificity against standard biomedical examinations.[30] Incident cardiovascular disease was denoted by atrial fibrillation (ICD-10 codes under I48.0, I48.1, I48.2, I48.3, I48.4, I48.9; ICD-9 codes 427.31, 427.32) stroke (ICD-10 codes under I60, I61, I63, and I64; ICD-9 codes 430, 431, 434, and 436), myocardial infarction (ICD-10 codes under I21; ICD-9 codes under 410), heart failure (ICD10-codes under I50), definite angina (ICD-10 codes under I20; ICD-9 codes under 410, both verified from medical records), peripheral artery disease (ICD-10 codes I70.2, I73.3, I73.9, I74.3-5, E10.5, E11.5, E12.5, E13.5, E14.5; ICD-9 codes 250.7, 440.2, 440.4, 443.8-9, 444.2, 444.81), coronary artery bypass graft, or percutaneous coronary intervention. In addition, ECG measurements in each 5-yeraly visits screened atrial fibrillation. Definition for diabetes in each 5-yearly visits was use of diabetes medication, fasting glucose ≥7.0 mmol/L, 2h post-load glucose ≥11.1 mmol/L, or Hba1c% ≥6.5%.

Imputation model

In the Whitehall II study no participants were missing protein measurements but some covariates were missing; 26% had missing education status, 11% were missing *APOE* and 10% BMI. Education variable was introduced to questionnaire during data collection meaning that it is likely to be missing at random. *APOE* values were measured in a random sample, suggesting values are also missing at random. BMI values are missing due to lack of funds to cover sickness absences of the research nurse collecting height and weight at phase 5 and are also likely to be missing at random. Percentages of participants missing data for other variables were very low and unlikely to affect the imputation results regardless of the missing data mechanism. For these reasons, we used the missing at random assumption and conducted multiple imputation for covariate-adjusted analyses. Our imputation model included age, sex, education, socioeconomic status, systolic blood pressure, total cholesterol, high-density lipoprotein cholesterol, antihypertensive medication, diabetes status, ApoE status, alcohol consumption, General Health Questionnaire, BMI, and smoking status. Continuous variables were imputed with linear regression, binary with logistic regression, categorical with ordered logistic regression, and right tailed integer data with negative binomial regression. Based on percentage of missing data and substantive model parameters from 100 iterations we chose to use 5 iterations and 30 imputations in the final analyses. We used an

imputation model based on substantive-model compatible fully conditional specification, which may be a better approach in survival analyses and in the presence of competing risks than the ordinary fully conditional model.[130]

The two-step approach used to identify protein-dementia association for replication

We used a two-step approach in Whitehall II to identify dementia-related proteins for replication analyses in the ARIC study. The first step was designed to filter out proteins with no or only weak association with cognitive decline rate after false discovery rate correction of 5%; we had a 80% statistical power to detect a beta of 0.08. The outcome, cognitive decline slope, is sensitive to any decrease in cognition, including both impairments that precede dementia and those not reaching levels that meet criteria for clinical dementia. The second step was therefore designed to identify from the proteins that passed the first step those that are also associated with dementia. We used a dementia outcome derived from electronic health records from the National Health Services (NHS) Hospital Episode Statistics (HES) database, an outcome with a high positive predictive value. The analysis model in the second step was the Cox proportional hazards model which had 80% power to identify a beta of 0.27 with a p-value<0.05. The replication analysis used the ARIC study, which used different dementia ascertainment methods, had a different dementia conversion rate, different participant characteristics at baseline, and originated from a different continent. Of the 21 proteins that passed first and second step in Whitehall II study, 15 (71%) were replicated in the ARIC study suggesting that our approach was successful within the limits of our dataset.

Supplementary Figures 1a and b. Sample size needed to detect a range of beta coefficients using linear regression or Cox proportional hazards model. Graph in part a is derived using sample size 2242 (dashed line), power 0.8, p-value 0.002, and standard deviation of 1 for both the covariate of interest and for the error term. Graph in part b is derived using outcome number of 106 (dashed line), power 0.8, p-value 0.05, and standard deviation 1 for covariate of interest.

a)

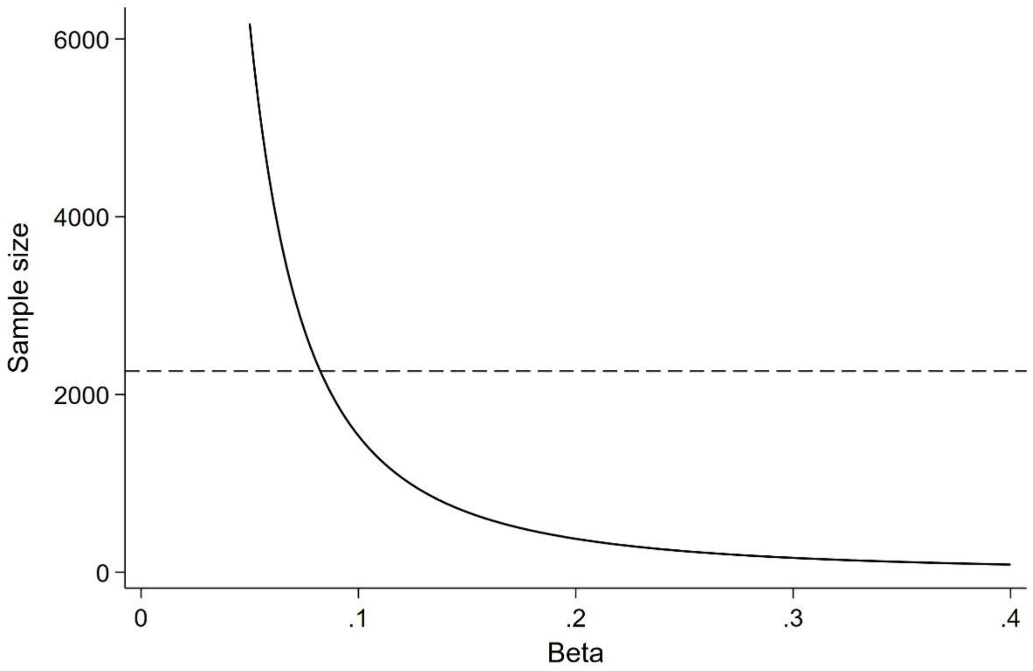

b)

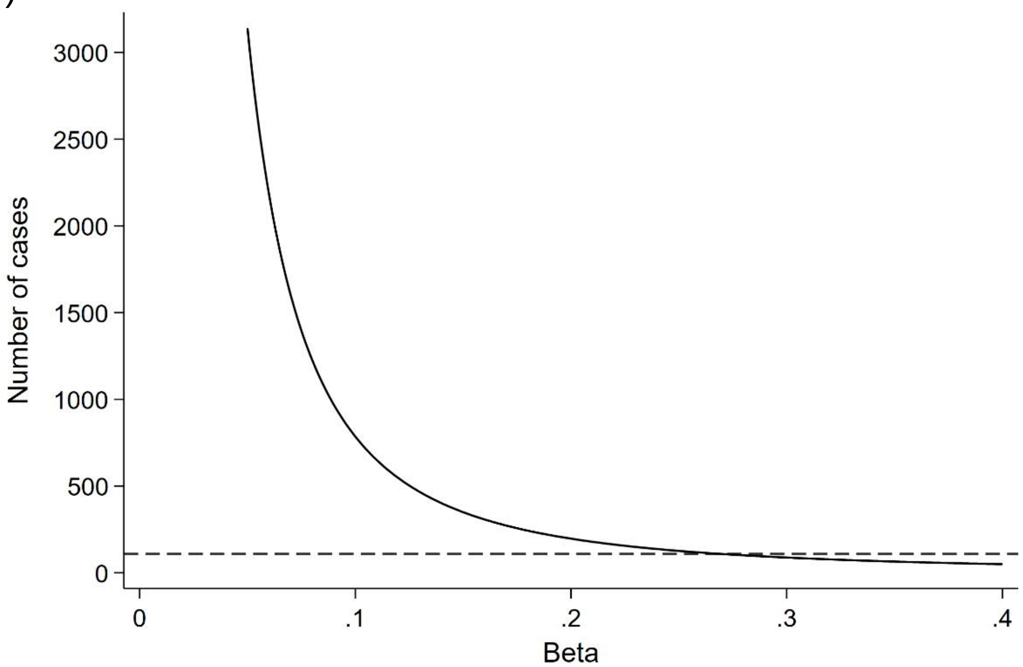

Supplementary Table 1. Beta for 1 standard deviation increase in amyloid, tau, and neurofilament related protein levels and cognitive decline. Results are from a linear regression model with cognitive decline slope as the outcome. The number of individuals included is 2,242. FDR, false discovery rate correction of 5%.

	Cognitive decline		
**Protein**	**Beta**	**FDR p-value**	**Original p-value**
**Amyloid, tau, and neurofilament related proteins**			
Amyloid-like protein 1	0.016	0.646	0.449
Amyloid-like protein 2	0.031	0.354	0.137
Serum amyloid A-1 protein	0.046	0.187	0.030
Serum amyloid A-2 protein	0.052	0.133	0.015
Serum amyloid A-4 protein	0.020	0.556	0.347
Serum amyloid P-component	0.021	0.528	0.318
Amyloid beta A4 protein	0.015	0.672	0.483
Amyloid beta A4 precursor protein-binding family B member 1	0.032	0.345	0.131
Amyloid beta A4 precursor protein-binding family B member 2	0.053	0.123	0.013
Amyloid beta A4 precursor protein-binding family B member 3	0.017	0.625	0.423
Apolipoprotein E	0.002	0.951	0.915
Microtubule-associated protein RP/EB family member 1	-0.011	0.749	0.589
Microtubule-associated protein RP/EB family member 2	-0.011	0.765	0.612
Microtubule-associated protein RP/EB family member 3	-0.017	0.627	0.426
Microtubule-associated proteins 1A/1B light chain 3A	0.029	0.382	0.168
Microtubule-associated proteins 1A/1B light chain 3B	0.008	0.822	0.698
Microtubule-associated protein tau	0.031	0.359	0.145
Regulator of microtubule dynamics protein 1	0.007	0.859	0.757
Regulator of microtubule dynamics protein 3	0.018	0.589	0.386
Janus kinase and microtubule-interacting protein 3	0.063	0.055	0.003
MAP/microtubule affinity-regulating kinase 3	0.004	0.916	0.851
Neurofilament light polypeptide	0.014	0.694	0.512
Neurofilament heavy polypeptide	0.044	0.200	0.036

Supplementary Figure 2. Associations between each of the 4953 proteins and rate of cognitive decline by –log10 p-values and beta coefficient. Inverse rank-based normal transformed proteins plotted by their betas and p-values from linear regression in which rate of cognitive decline was the outcome. The 15 proteins associated with cognitive decline and dementia in Whitehall II study and with dementia in the ARIC study are labelled.

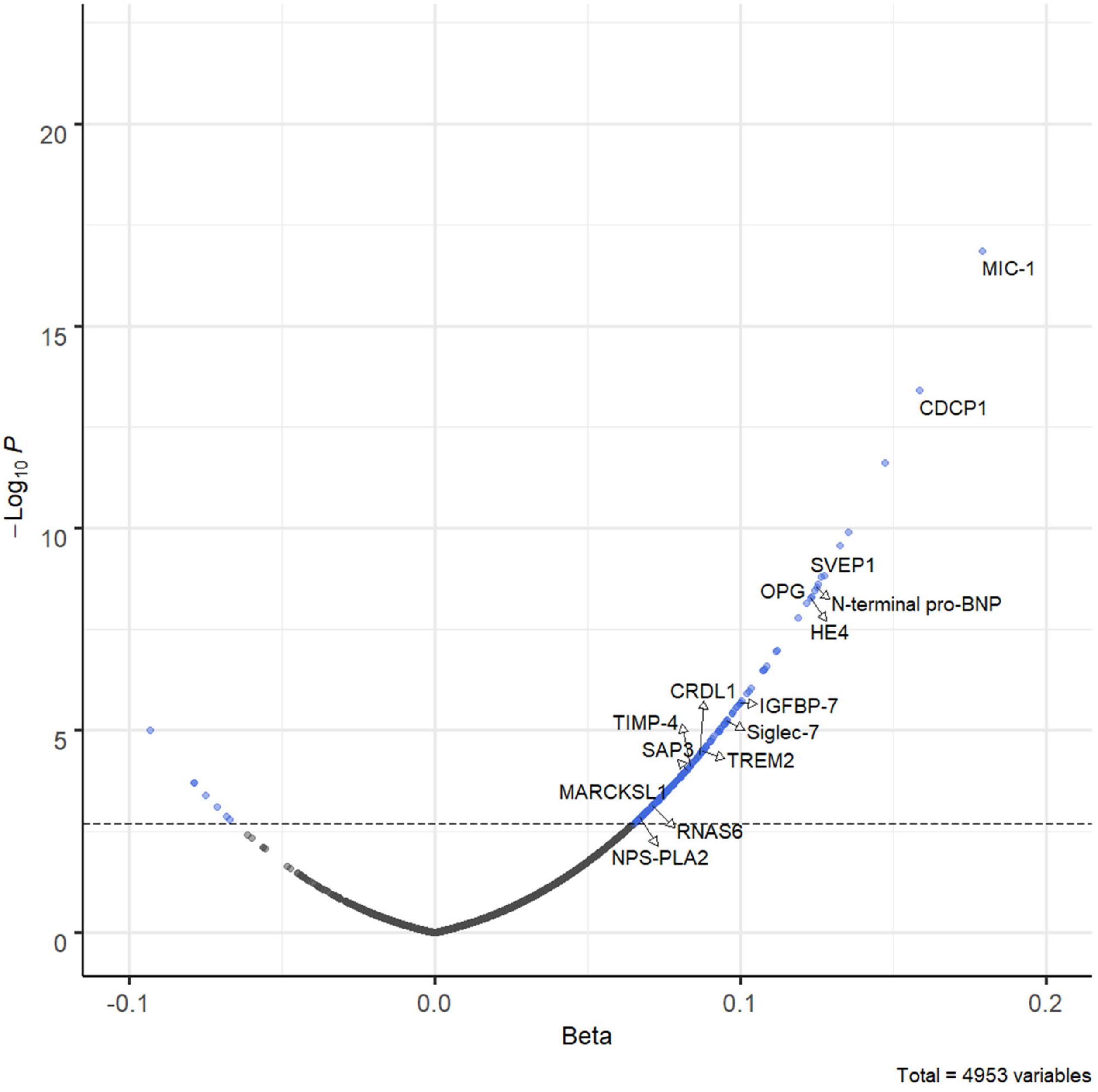

Supplementary Figure 3. Associations between each of the 4953 proteins and dementia by –log10 p-values and beta coefficient. Inverse rank-based normal transformed proteins plotted by their betas and p-values from Cox proportional hazards model in which binary dementia was the outcome. The 15 proteins that associated with cognitive decline and dementia in Whitehall II study and with dementia in the ARIC study are labelled.

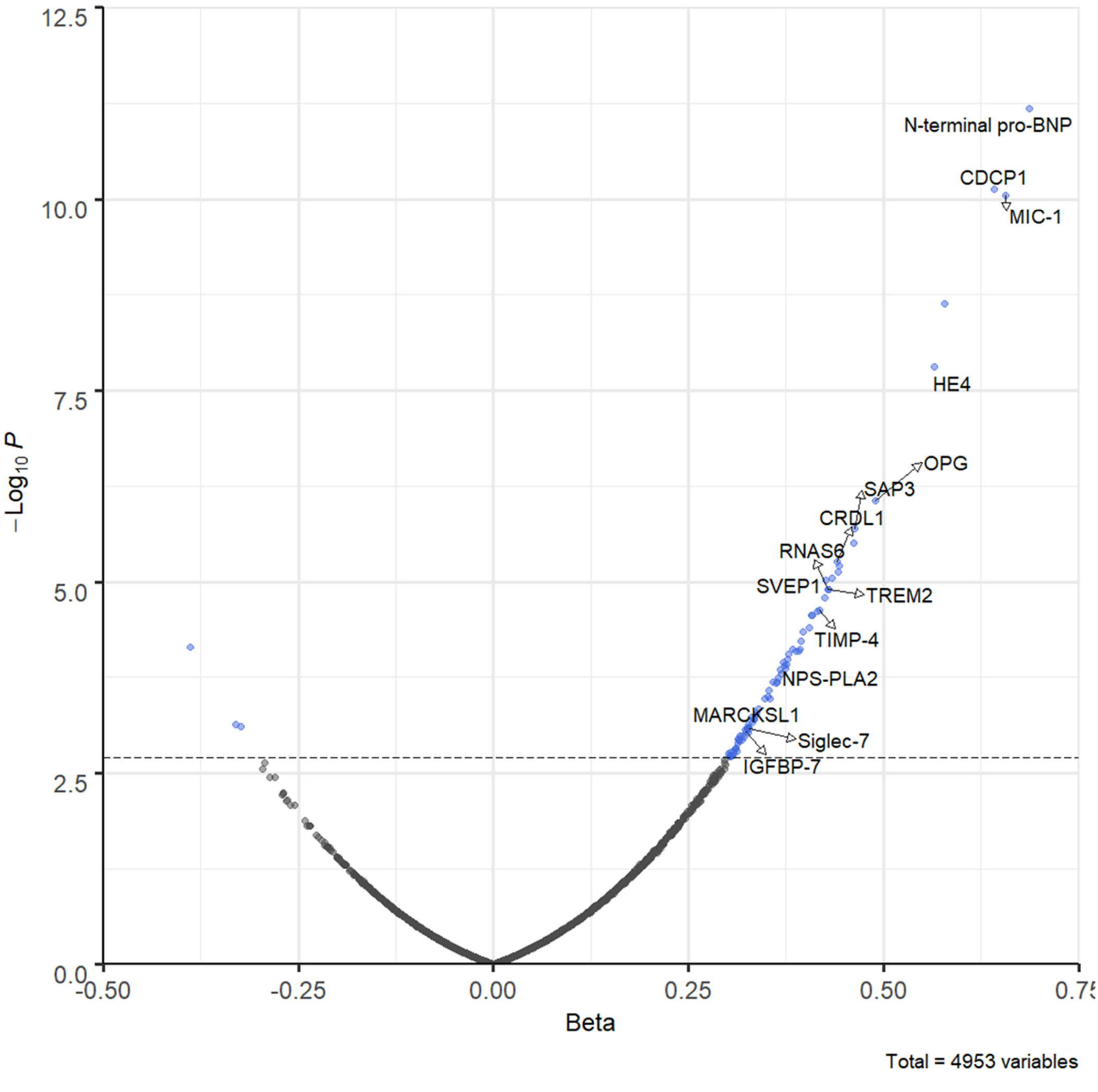

Supplementary Figure 4. Violin plots describing differences in standardized protein levels by dementia status. All the 15 inverse rank-based normal transformed proteins were elevated in those who developed dementia. Blue, no dementia. Green, dementia.

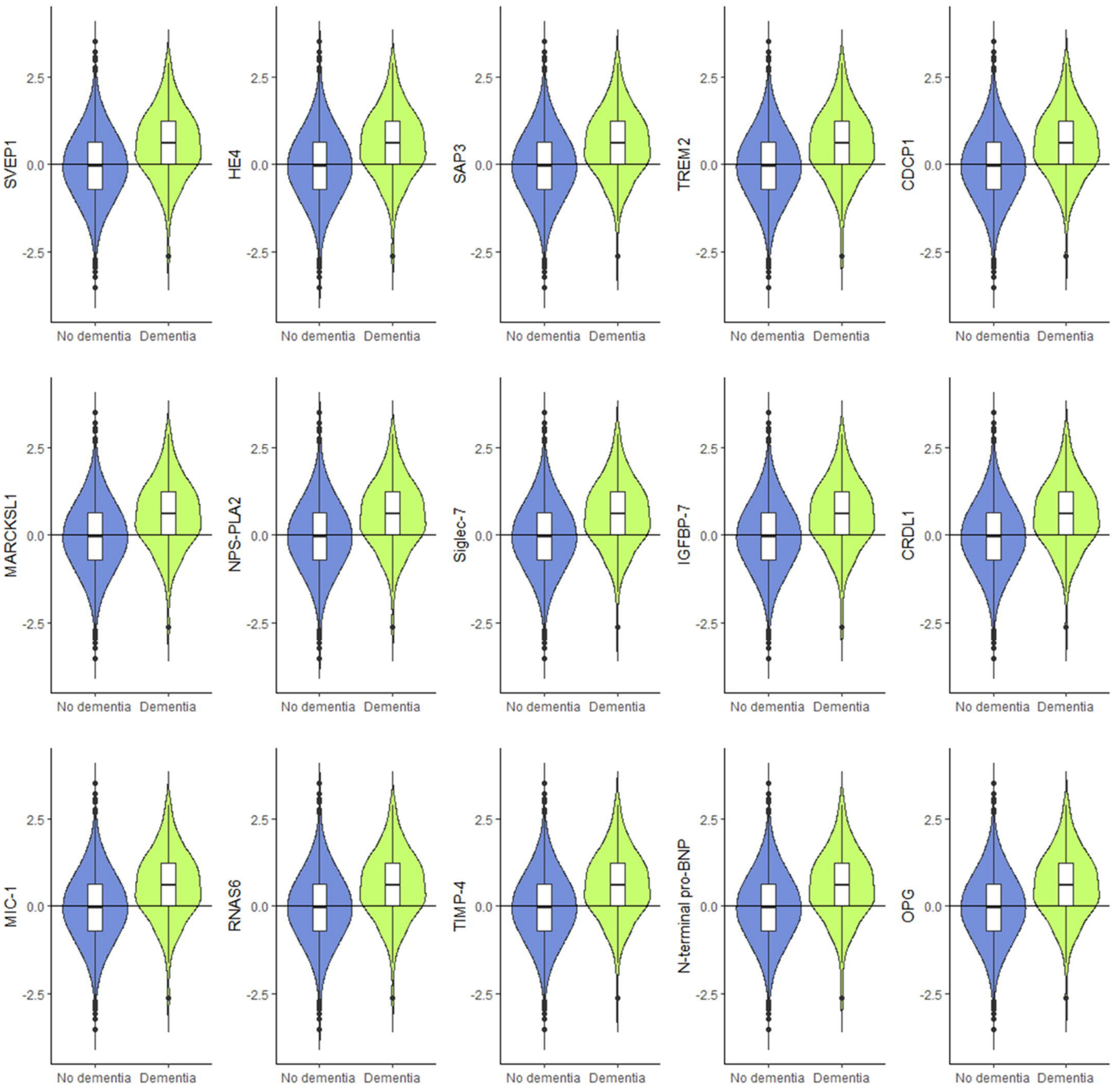

Supplementary Table 2. Associations between the cognitive decline slope and a SD increase in the 15 proteins that survived replication. Results are from a linear regression model with cognitive decline rate as the outcome. The number of individuals included is 2,242.

Protein	HR (95% CIs)	p-value	FDR corrected p-value
N-terminal pro-BNP	0.12 (0.08 to 0.17)	<0.001	<0.001
CDCP1	0.16 (0.12 to 0.20)	<0.001	<0.001
MIC-1	0.18 (0.14 to 0.22)	<0.001	<0.001
CRDL1	0.09 (0.05 to 0.13)	<0.001	0.003
RNAS6	0.07 (0.03 to 0.11)	0.001	0.023
SAP3	0.08 (0.04 to 0.12)	<0.001	0.006
HE4	0.12 (0.08 to 0.16)	<0.001	<0.001
TIMP-4	0.08 (0.04 to 0.13)	<0.001	0.005
IGFBP-7	0.10 (0.06 to 0.14)	<0.001	<0.001
OPG	0.12 (0.08 to 0.16)	<0.001	<0.001
Siglec-7	0.10 (0.05 to 0.14)	<0.001	0.001
SVEP1	0.13 (0.09 to 0.17)	<0.001	<0.001
TREM2	0.09 (0.05 to 0.13)	<0.001	0.003
NPS-PLA2	0.07 (0.03 to 0.11)	0.001	0.035
MARCKSL1	0.07 (0.03 to 0.11)	0.001	0.021

Supplementary Table 3. Associations between the decline slopes in all cognitive domains measured and a SD increase in the 15 proteins that survived replication. Results are from a linear regression model with cognitive domain decline rate as the outcome. The number of individuals included is 2,242.

	**Executive function**		**Phonemic fluency**	
**Protein**	**HR (95% CIs)**	**p-value**	**HR (95% CIs)**	**p-value**
N-terminal pro-BNP	0.14 (0.10 to 0.18)	<0.001	0.10 (0.06 to 0.14)	<0.001
CDCP1	0.17 (0.12 to 0.21)	<0.001	0.08 (0.04 to 0.12)	<0.001
MIC-1	0.20 (0.16 to 0.24)	<0.001	0.08 (0.04 to 0.12)	<0.001
CRDL1	0.08 (0.04 to 0.12)	<0.001	0.04 (0.00 to 0.08)	0.046
RNAS6	0.10 (0.06 to 0.14)	<0.001	0.03 (-0.02 to 0.07)	0.225
SAP3	0.10 (0.06 to 0.14)	<0.001	0.04 (0.00 to 0.08)	0.058
HE4	0.15 (0.10 to 0.19)	<0.001	0.05 (0.01 to 0.09)	0.013
TIMP-4	0.11 (0.07 to 0.15)	<0.001	0.06 (0.02 to 0.10)	0.005
IGFBP-7	0.06 (0.02 to 0.10)	0.005	0.07 (0.02 to 0.11)	0.002
OPG	0.14 (0.10 to 0.18)	<0.001	0.05 (0.01 to 0.09)	0.021
Siglec-7	0.07 (0.03 to 0.11)	0.001	0.03 (-0.01 to 0.07)	0.134
SVEP1	0.13 (0.09 to 0.17)	<0.001	0.09 (0.05 to 0.13)	<0.001
TREM2	0.09 (0.05 to 0.13)	<0.001	0.06 (0.02 to 0.11)	0.002
NPS-PLA2	0.09 (0.05 to 0.13)	<0.001	0.02 (-0.02 to 0.06)	0.328
MARCKSL1	0.09 (0.04 to 0.13)	<0.001	0.06 (0.02 to 0.10)	0.007

	**Memory**		**Semantic fluency**	
**Protein**	**HR (95% CIs)**	**p-value**	**HR (95% CIs)**	**p-value**
N-terminal pro-BNP	0.01 (-0.03 to 0.05)	0.734	0.04 (0.00 to 0.08)	0.056
CDCP1	0.04 (0.00 to 0.08)	0.050	0.04 (0.00 to 0.08)	0.055
MIC-1	0.04 (0.00 to 0.08)	0.064	0.06 (0.01 to 0.10)	0.008
CRDL1	0.03 (-0.01 to 0.07)	0.197	0.03 (-0.01 to 0.07)	0.134
RNAS6	-0.01 (-0.05 to 0.03)	0.633	0.01 (-0.03 to 0.05)	0.595
SAP3	-0.02 (-0.06 to 0.02)	0.356	0.05 (0.01 to 0.09)	0.022
HE4	0.00 (-0.04 to 0.04)	0.879	0.03 (-0.01 to 0.07)	0.121
TIMP-4	0.02 (-0.02 to 0.06)	0.325	-0.01 (-0.05 to 0.04)	0.794
IGFBP-7	0.06 (0.02 to 0.10)	0.005	0.03 (-0.01 to 0.07)	0.200
OPG	0.03 (-0.01 to 0.07)	0.132	0.04 (0.00 to 0.08)	0.068
Siglec-7	0.06 (0.01 to 0.10)	0.009	0.02 (-0.02 to 0.07)	0.256
SVEP1	0.02 (-0.02 to 0.07)	0.245	0.04 (0.00 to 0.08)	0.083
TREM2	0.01 (-0.04 to 0.05)	0.786	0.02 (-0.03 to 0.06)	0.469
NPS-PLA2	0.03 (-0.01 to 0.08)	0.108	0.00 (-0.04 to 0.04)	0.897
MARCKSL1	0.02 (-0.02 to 0.06)	0.429	0.03 (-0.01 to 0.07)	0.192

Supplementary Table 4. Protein expression levels in the brain tissue for 15 proteins that survived replication. The expression levels are derived from the Human Protein Atlas.

Protein	Expression in brain
N-terminal pro-BNP	Not known
CDCP1	Low
MIC-1	Not detected
CRDL1	Not known
RNAS6	Not detected
SAP3	High
HE4	Low
TIMP-4	Not known
IGFBP-7	Not known
OPG	Not known
SIGLEC-7	Not known
SVEP1	Low
TREM2	Medium
NPS-PLA2	Not detected
MARCKSL1	Medium

Supplementary Table 5. Sensitivity analyses for the 15 proteins associated with both cognitive decline and dementia in the Whitehall II study. The analyses include 2,262 individuals, 106 dementia cases and a mean follow-up of 20 years.

	Model 1		Model 2		Model 3	
**Protein name**	HR (95% CIs)	p-value	HR (95% CIs)	p-value	HR (95% CIs)	p-value
N-terminal pro-BNP	1.52 (1.24 to 1.88)	<0.001	1.49 (1.21 to 1.85)	<0.001	1.56 (1.26 to 1.94)	<0.001
CDCP1	1.42 (1.16 to 1.73)	<0.001	1.43 (1.17 to 1.76)	0.001	1.38 (1.13 to 1.70)	0.002
MIC-1	1.31 (1.04 to 1.65)	0.021	1.30 (1.02 to 1.65)	0.031	1.31 (1.03 to 1.67)	0.029
CRDL1	1.30 (1.06 to 1.59)	0.010	1.27 (1.04 to 1.55)	0.021	1.30 (1.06 to 1.60)	0.011
RNAS6	1.30 (1.07 to 1.57)	0.007	1.28 (1.05 to 1.57)	0.014	1.30 (1.06 to 1.59)	0.011
SAP3	1.29 (1.06 to 1.57)	0.012	1.28 (1.04 to 1.57)	0.019	1.29 (1.05 to 1.58)	0.016
HE4	1.29 (1.04 to 1.60)	0.019	1.34 (1.07 to 1.69)	0.012	1.34 (1.06 to 1.69)	0.015
TIMP-4	1.29 (1.05 to 1.59)	0.017	1.24 (1.01 to 1.53)	0.042	1.24 (1.00 to 1.54)	0.051
IGFBP-7	1.27 (1.05 to 1.54)	0.015	1.22 (1.01 to 1.49)	0.044	1.25 (1.03 to 1.53)	0.027
OPG	1.26 (1.02 to 1.56)	0.032	1.26 (1.02 to 1.56)	0.030	1.27 (1.03 to 1.58)	0.029
SIGLEC-7	1.25 (1.03 to 1.52)	0.025	1.23 (1.00 to 1.52)	0.054	1.22 (0.99 to 1.50)	0.068
SVEP1	1.24 (1.02 to 1.51)	0.032	1.20 (0.98 to 1.47)	0.077	1.24 (1.01 to 1.52)	0.036
TREM2	1.24 (1.01 to 1.51)	0.037	1.23 (1.00 to 1.50)	0.046	1.25 (1.02 to 1.54)	0.032
NPS-PLA2	1.24 (1.01 to 1.51)	0.038	1.20 (0.98 to 1.48)	0.083	1.24 (1.00 to 1.53)	0.049
MARCKSL1	1.22 (1.01 to 1.48)	0.041	1.20 (0.99 to 1.46)	0.069	1.19 (0.98 to 1.45)	0.084

	Model 4		Model 5	
**Protein name**	HR (95% CIs)	p-value	HR (95% CIs)	p-value
N-terminal pro-BNP	1.53 (1.24 to 1.89)	<0.001	1.47 (1.18 to 1.83)	0.001
CDCP1	1.41 (1.15 to 1.73)	0.001	1.39 (1.13 to 1.71)	0.002
MIC-1	1.31 (1.04 to 1.65)	0.021	1.24 (0.98 to 1.57)	0.069
CRDL1	1.30 (1.07 to 1.59)	0.010	1.26 (1.03 to 1.55)	0.027
RNAS6	1.30 (1.08 to 1.57)	0.006	1.27 (1.04 to 1.54)	0.016
SAP3	1.29 (1.06 to 1.57)	0.011	1.28 (1.05 to 1.57)	0.017
HE4	1.29 (1.04 to 1.60)	0.019	1.27 (1.02 to 1.57)	0.032
TIMP-4	1.29 (1.05 to 1.59)	0.017	1.25 (1.01 to 1.55)	0.040
IGFBP-7	1.27 (1.05 to 1.54)	0.014	1.22 (1.00 to 1.49)	0.045
OPG	1.27 (1.02 to 1.57)	0.030	1.22 (0.99 to 1.51)	0.067
SIGLEC-7	1.25 (1.03 to 1.52)	0.023	1.22 (1.00 to 1.49)	0.054
SVEP1	1.24 (1.02 to 1.51)	0.030	1.19 (0.98 to 1.46)	0.084
TREM2	1.24 (1.01 to 1.51)	0.039	1.24 (1.01 to 1.52)	0.043
NPS-PLA2	1.24 (1.01 to 1.51)	0.035	1.23 (1.01 to 1.51)	0.043
MARCKSL1	1.22 (1.01 to 1.48)	0.040	1.20 (0.99 to 1.47)	0.064

Model 1 is adjusted for age, sex, and ethnicity in the Whitehall II study
Model 2 is multiple imputation model adjusted for age, sex, ethnicity, systolic blood pressure, total cholesterol, antihypertensive medication, smoking, diabetes, *APOE* genotype, BMI, alcohol consumption, education, and GHQ score
Model 3 is as model 2 but the first 10 years of follow-up has been excluded
Model 4 is as model 2 but controls for competing risk of death
Model 5 is as model 1 and additionally adjusted for stroke, heart failure, coronary heart disease, and atrial fibrillation, treated as time-varying covariates

Supplementary Table 6. Sensitivity analyses including all or only white participants of the ARIC study and the 15 proteins associated with both cognitive decline and dementia in the Whitehall II study. The associations for all are from age, sex, and race-study center adjusted Cox proportional hazards regression with 11,395 individuals, 1,942 dementia cases and 17 years of follow up. The second model includes white participants only and is age, sex, and study center adjusted Cox proportional hazards regression with 8,991 individuals, 1,438 dementia cases and 17 years of follow up.

	All		White participants only	
**Protein name**	HR (95% CIs)	p-value	HR (95% CIs)	p-value
N-terminal pro-BNP	1.08 (1.03 to 1.13)	<0.001	1.04 (0.98 to 1.10)	0.233
CDCP1	1.26 (1.17 to 1.37)	<0.001	1.20 (1.09 to 1.32)	<0.001
MIC-1	1.64 (1.49 to 1.82)	<0.001	1.67 (1.49 to 1.88)	<0.001
CRDL1	1.20 (1.01 to 1.42)	0.039	1.20 (0.99 to 1.47)	0.068
RNAS6	1.23 (1.11 to 1.37)	<0.001	1.18 (1.05 to 1.34)	0.007
SAP3	1.32 (1.12 to 1.55)	0.001	1.28 (1.05 to 1.57)	0.015
HE4	1.44 (1.28 to 1.62)	<0.001	1.40 (1.22 to 1.62)	<0.001
TIMP-4	1.12 (1.01 to 1.26)	0.039	1.17 (1.03 to 1.34)	0.018
IGFBP-7	1.43 (1.18 to 1.72)	<0.001	1.37 (1.10 to 1.70)	0.005
OPG	1.34 (1.20 to 1.50)	<0.001	1.39 (1.22 to 1.60)	<0.001
SIGLEC-7	1.24 (1.06 to 1.45)	0.008	1.41 (1.17 to 1.69)	<0.001
SVEP1	1.39 (1.23 to 1.58)	<0.001	1.33 (1.14 to 1.54)	<0.001
TREM2	1.13 (1.04 to 1.23)	0.003	1.15 (1.03 to 1.27)	0.010
NPS-PLA2	1.13 (1.03 to 1.25)	0.008	1.20 (1.07 to 1.35)	0.002
MARCKSL1	1.11 (1.01 to 1.22)	0.035	1.03 (0.92 to 1.15)	0.612
SPONDIN-1	1.18 (1.00 to 1.40)	0.055	1.05 (0.86 to 1.29)	0.615
IGFBP-2	1.06 (0.99 to 1.13)	0.089	1.00 (0.93 to 1.08)	0.932
NEUROPEPTIDE W	1.03 (1.00 to 1.07)	0.088	1.00 (0.96 to 1.04)	0.963
ST4S6	0.96 (0.78 to 1.18)	0.721	0.93 (0.73 to 1.19)	0.554
TPPP2	1.04 (0.93 to 1.17)	0.459	1.00 (0.88 to 1.14)	0.984
LEAP-1	1.02 (0.97 to 1.06)	0.483	1.02 (0.97 to 1.07)	0.533

Supplementary Table 7. Associations between common dementia risk factors and dementia in Whitehall II, and estimates from most recent meta-analyses. The results are from Cox proportional hazards model adjusted for age and sex. The mean follow-up is 20 years, total number of participants is between 1,950 and 2,262 and cases between 89 and 106 due to missing data in BMI and *APOE* variables. Reference 47, Sipilä, P. N. et al. Long-term risk of dementia following hospitalization due to physical diseases: A multicohort study. Alzheimers Dement. 16, 1686-1695 (2020). Reference 48, Stocker, H. et al. Prediction of clinical diagnosis of Alzheimer's disease, vascular, mixed, and all-cause dementia by a polygenic risk score and APOE status in a community-based cohort prospectively followed over 17 years. Mol. Psychiatry (2020).

Risk Factor	Whitehall II	Low estimate	High estimate
Hypertension	1.56 (1.04 - 2.33)	1.18 (1.02 - 1.35) [47]	1.61 (1.16 - 2.24) [47]
Obesity (BMI ≥30)	1.86 (1.18 - 2.94)	0.91 (0.83 - 1.01) [47]	1.91 (1.40 - 2.62) [47]
Smoking	1.13 (0.59 - 2.17)	1.27 (1.02 - 1.60) [47]	1.30 (1.18 - 1.45) [47]
Depression (GHQ ≥5)	1.30 (0.83 - 2.03)	1.08 (1.04 - 1.12) [47]	1.98 (1.50 - 2.63) [47]
Physical inactivity	0.83 (0.53 - 1.28)	1.01 (0.89 - 1.13) [47]	1.39 (1.16 - 1.67) [47]
Diabetes	1.24 (0.77 - 2.00)	1.43 (1.33 - 1.53) [47]	1.73 (1.65 - 1.82) [47]
Apolipoprotein E, 1 or 2 ε4 alleles	2.05 (1.34 - 3.15)	2.60 (1.60 - 4.00) [48]	3.34 (2.24 - 4.99) [48]

Supplementary Figure 5. Between protein correlations. Correlations are Spearman correlations and were all positive and ≤|0.53|.

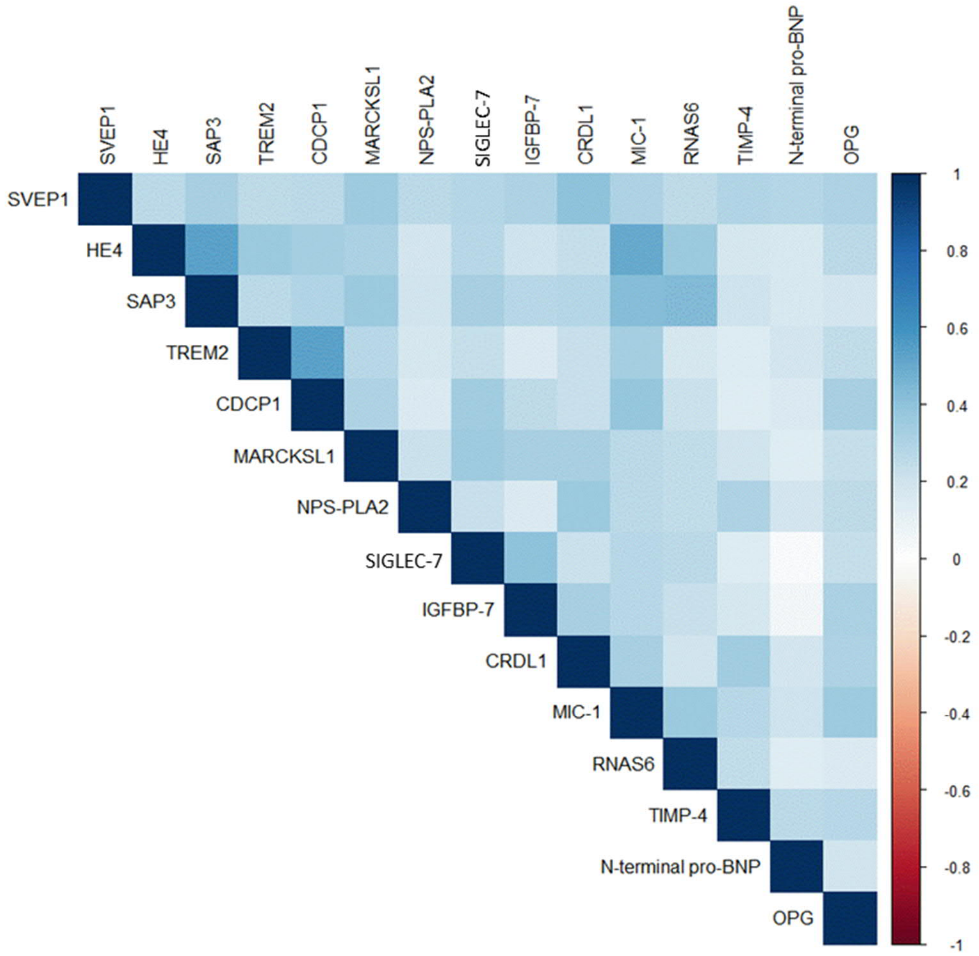

Supplementary Table 8. Sensitivity analyses of dementia subtypes for the 15 proteins associated with both cognitive decline and dementia in the Whitehall II study. The associations are from age, sex, and ethnicity adjusted Cox proportional hazards model with 2,262 individuals, mean follow-up of 20 years, and 72 dementias with comorbid vascular disease or 34 dementias without comorbid vascular disease.

	Dementia with comorbid vascular disease		Dementia without comorbid vascular disease		
**Protein name**	HR (95% CIs)	p-value	HR (95% CIs)	p-value	p-value for difference
N-terminal pro-BNP	1.44 (1.11 to 1.86)	0.006	1.74 (1.20 to 2.52)	0.004	0.392
CDCP1	1.43 (1.11 to 1.83)	0.005	1.36 (0.95 to 1.96)	0.096	0.919
MIC-1	1.23 (0.93 to 1.63)	0.147	1.41 (0.93 to 2.15)	0.104	0.377
CRDL1	1.27 (0.99 to 1.63)	0.057	1.34 (0.94 to 1.92)	0.105	0.816
RNAS6	1.27 (1.01 to 1.61)	0.044	1.35 (0.96 to 1.89)	0.081	0.606
SAP3	1.21 (0.95 to 1.54)	0.127	1.50 (1.05 to 2.14)	0.026	0.257
HE4	1.28 (0.99 to 1.67)	0.061	1.26 (0.86 to 1.85)	0.238	0.861
TIMP-4	1.31 (1.01 to 1.69)	0.044	1.16 (0.80 to 1.69)	0.426	0.806
IGFBP-7	1.27 (1.00 to 1.60)	0.050	1.28 (0.90 to 1.80)	0.166	0.856
OPG	1.16 (0.89 to 1.50)	0.277	1.47 (1.00 to 2.17)	0.051	0.241
SIGLEC-7	1.27 (1.00 to 1.61)	0.049	1.23 (0.87 to 1.74)	0.251	0.809
SVEP1	1.17 (0.92 to 1.49)	0.203	1.36 (0.96 to 1.93)	0.085	0.499
TREM2	1.30 (1.01 to 1.66)	0.041	1.14 (0.80 to 1.63)	0.473	0.554
NPS-PLA2	1.27 (0.99 to 1.62)	0.057	1.25 (0.87 to 1.78)	0.226	0.903
MARCKSL1	1.17 (0.92 to 1.48)	0.205	1.35 (0.95 to 1.90)	0.091	0.512

Supplementary Table 9. Spearman correlations between plasma proteins measures with SOMAscan version 4 and Olink Explore platform. RNAS6, SAP3, SVEP1, and MARCKSL1 were not available in Olink Explore platform.

Protein	Correlation
N-terminal pro-BNP	0.46
CDCP1	0.87
MIC-1	0.84
CRDL1	0.57
RNAS6	N/A
SAP3	N/A
HE4	0.85
TIMP-4	0.86
IGFBP-7	0.65
OPG	0.83
Siglec-7	0.69
SVEP1	N/A
TREM2	0.84
NPS-PLA2	0.76
MARCKSL1	N/A

Supplementary Table 10. Validation of Somalogic aptamer-based protein measurements. DDA-MS = Data-dependent acquisition Tandem Mass Spectrometry, cis pQTL = cis protein quantitative loci, MRM-MS = Multiple reaction monitoring Tandem Mass Spectrometry, Indirect = association observed between MRM-MS measured SAP3 and Alzheimer's diseases. [10,20,25-28]

Full aptamer target name	Aptamer target	Entrez Gene Symbol	Method of validation	Other comments from Somalogic
N-terminal pro-BNP	N-terminal pro-BNP	NPPB	cis pQTL data,[10,25] Olink panel,[25] sandwich immunoassay[26]	
CUB domain-containing protein 1	CDCP1	CDCP1	cis pQTL data[10]	
Growth/differentiation factor 15	MIC-1	GDF15	cis pQTL data,[10,25] Olink panel,[25] sandwich immunoassay[27]	
Chordin-like protein 1	CRDL1	CHRDL1	DDA-MS Data[10]	
Ribonuclease K6	RNAS6	RNASE6	cis pQTL data[10,25]	
Ganglioside GM2 activator	SAP3	GM2A	Indirect MRM-MS Data[28]	
WAP four-disulfide core domain protein 2	HE4	WFDC2	DDA-MS Data,[10] cis pQTL data[10]	
Metalloproteinase inhibitor 4	TIMP-4	TIMP4	cis pQTL data,[10,25] Olink panel[25]	
Insulin-like growth factor-binding protein 7	IGFBP-7	IGFBP7	DDA-MS Data,[10] cis pQTL data,[10,25] Olink panel[25]	
Tumor necrosis factor receptor superfamily member 11B	OPG	TNFRSF11B	cis pQTL data[10]	No binding was observed with Tumor necrosis factor receptor superfamily member 6B or 1B.
Sialic acid-binding Ig-like lectin 7	Siglec-7	SIGLEC7	cis pQTL data[10,25]	No binding was observed with Sialic acid-binding Ig-like lectin 6, 8, or 9
Sushi, von Willebrand factor type A, EGF and pentraxin domain-containing protein 1	SVEP1	SVEP1	MRM-MS Data,[10] DDA-MS Data,[10] cis pQTL data[10,25]	
Triggering receptor expressed on myeloid cells 2	TREM2	TREM2	MRM-MS Data,[10] indirect sandwich immunoassay[56]	
Phospholipase A2, membrane associated	NPS-PLA2	PLA2G2A	DDA-MS Data,[10] cis pQTL data[10,25]	
MARCKS-related protein	MARCKSL1	MARCKSL1	-	

Supplementary Figure 6. Hypothesize dementia-related pathologies for 15 proteins associated with cognitive decline rate and dementia risk. A schematic overview of the potential processes linking the proteins with **a.** increased innate and **b.** adaptive immunity responses in blood circulation and the brain, **c.** dysfunction of the blood-brain barrier, **d.** atherosclerotic and thrombotic changes in cerebral capillaries, and **e.** central insulin resistance in the brain. BBB, blood brain barrier. CNS, central nervous system. GLUT4, Glucose transporter type 4. IGF-1, Insulin-like growth factor 1. IGF-2, Insulin-like growth factor 2. NK-cells, natural killer cells. NO, nitric oxide.

a) Innate immunity

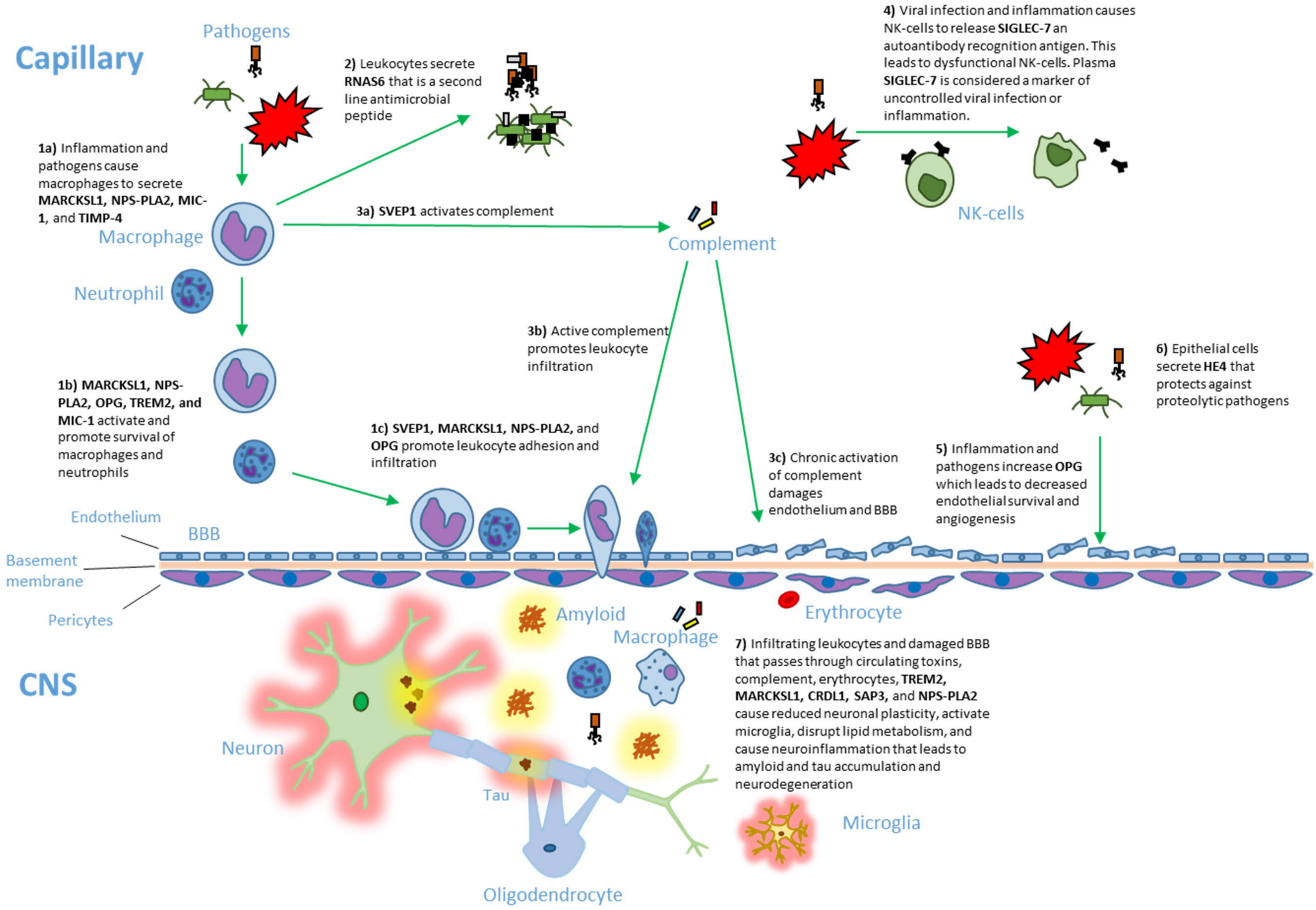

b) Adaptive immunity

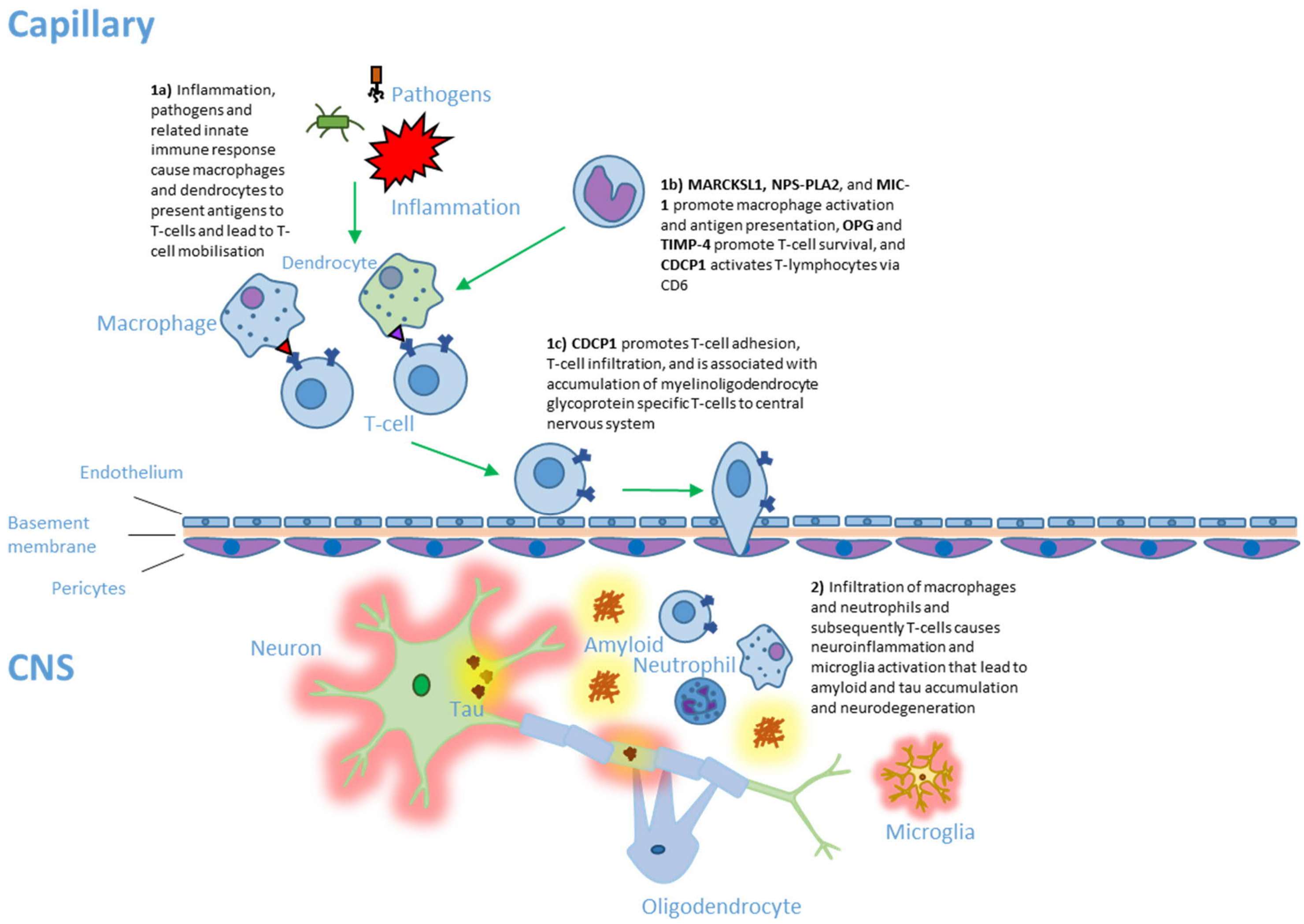

c) Blood brain barrier breakdown

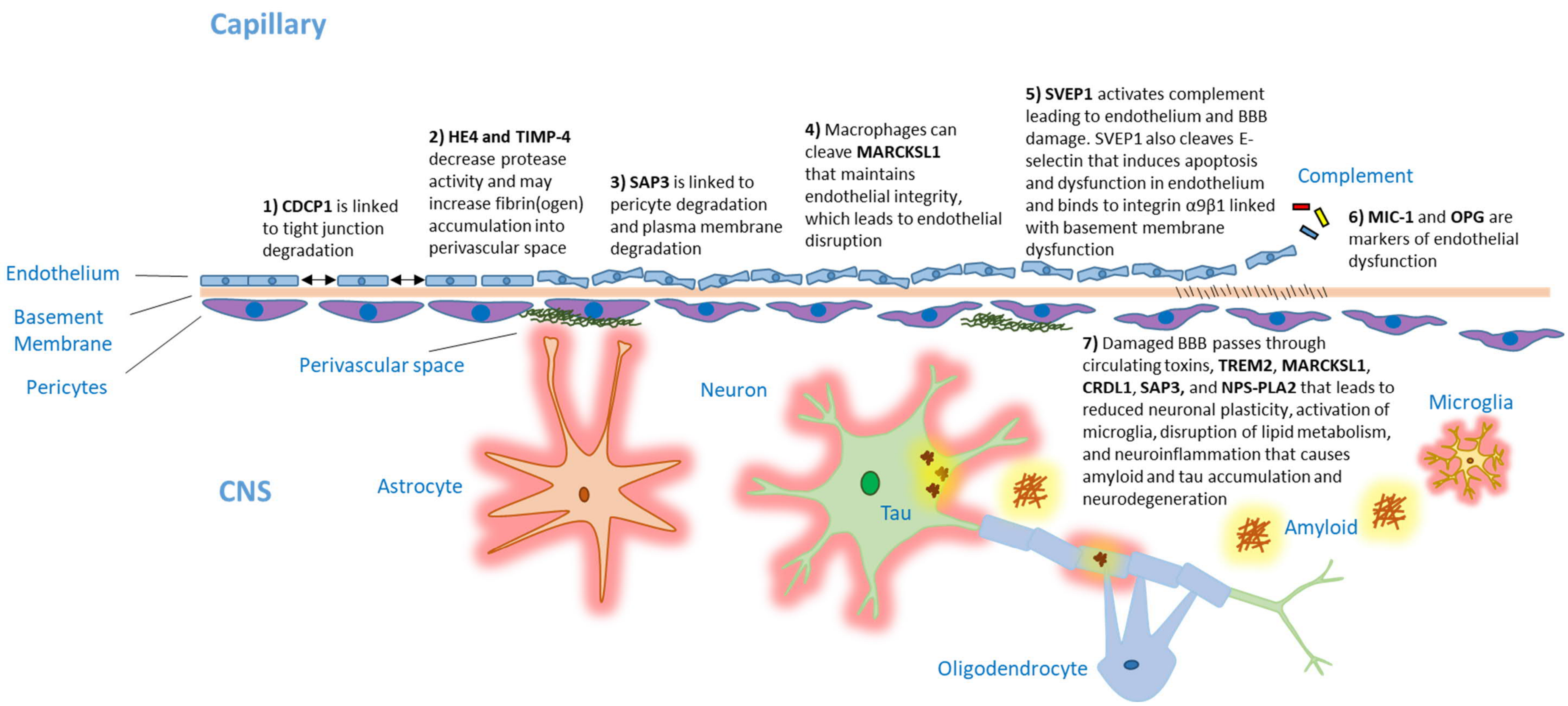

d) Atherosclerosis and thrombosis

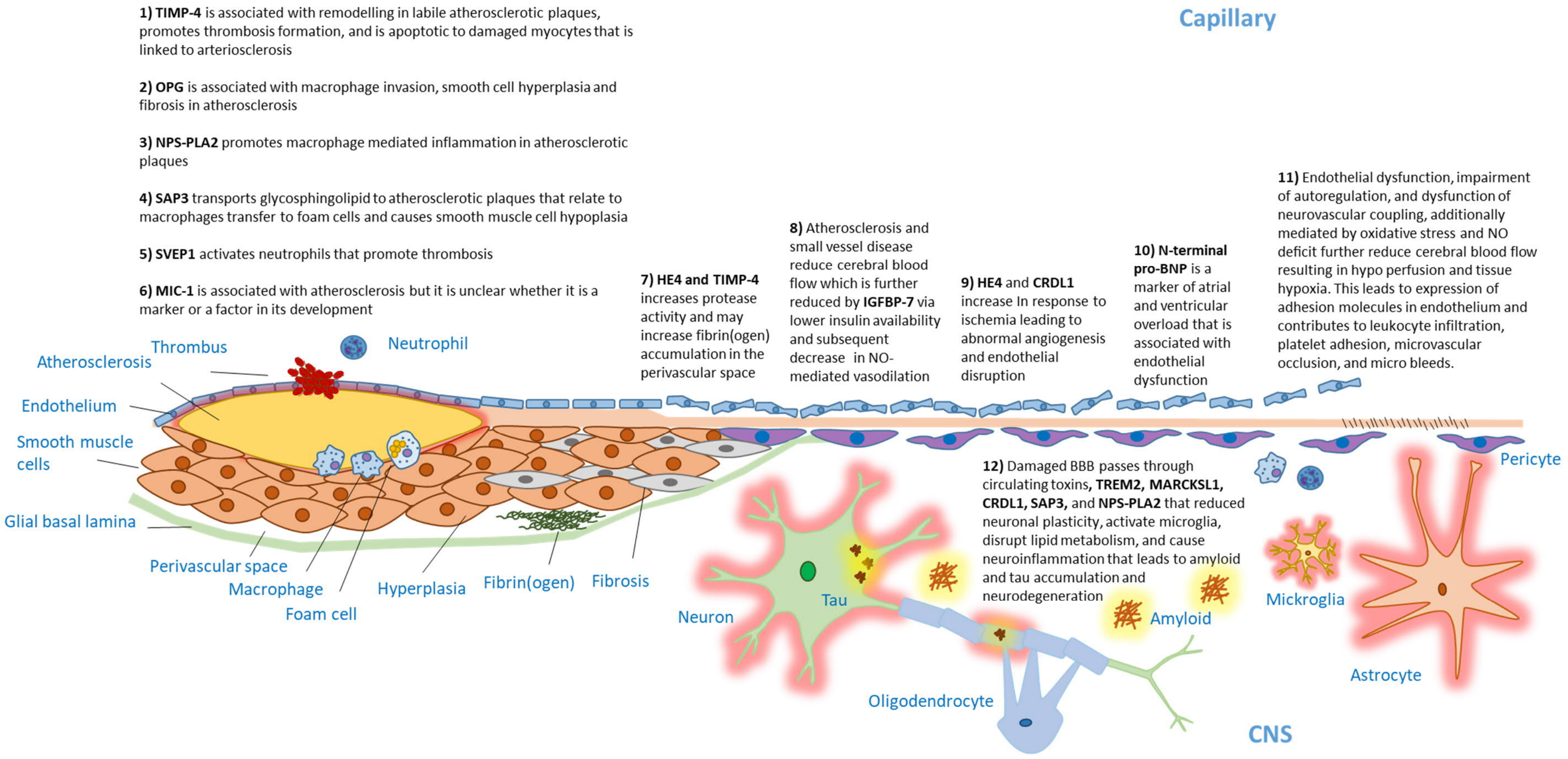

e) Central insulin resistance

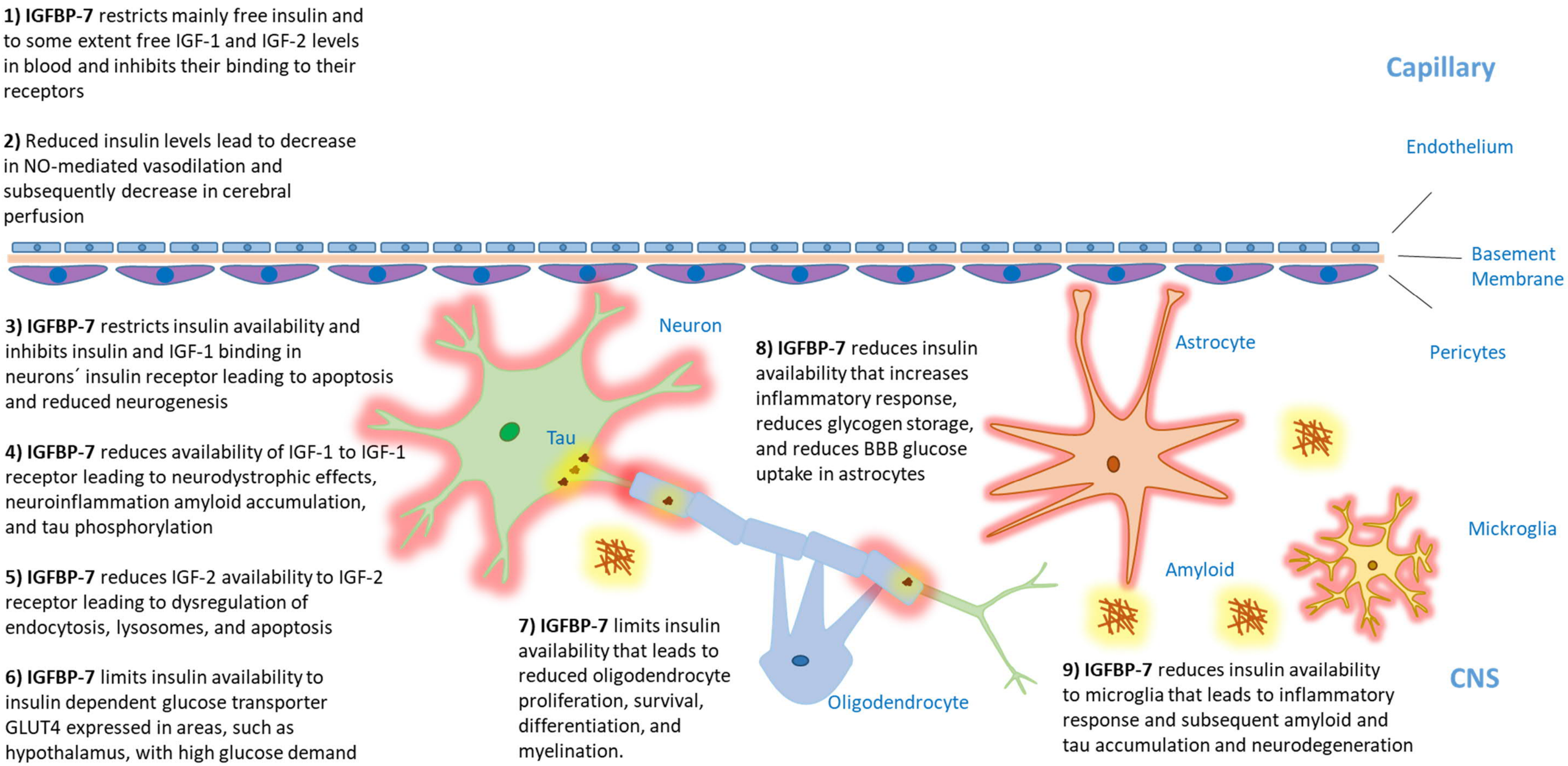

Supplement: Supplementary file 1 — Supporting Information [file ALZ-18-612-s001.pdf]
